# CXCR4 blockade reduces the severity of murine heart allograft rejection by plasmacytoid dendritic cell-mediated immune regulation

**DOI:** 10.1038/s41598-021-03115-z

**Published:** 2021-12-10

**Authors:** Jian Fu, Christian H. K. Lehmann, Xinning Wang, Mandy Wahlbuhl, Ida Allabauer, Benjamin Wilde, Lukas Amon, Sebastian Dolff, Robert Cesnjevar, Andreas Kribben, Joachim Woelfle, Wolfgang Rascher, Peter F. Hoyer, Diana Dudziak, Oliver Witzke, André Hoerning

**Affiliations:** 1 Department of Nephrology, University Hospital Essen, University Duisburg-Essen, Essen, Germany; 2 Department for Pediatric and Adolescent Medicine, University Hospital Erlangen, Friedrich-Alexander University Erlangen-Nürnberg, Loschgestrasse 15, 91054 Erlangen, Germany; 3 The Emergency and Trauma Center, The First Affiliated Hospital of Hai Nan Medical University, Haikou, China; 4 Department of Dermatology, University Hospital Erlangen, Friedrich-Alexander University of Erlangen-Nürnberg, Research Module II, Hartmannstr. 14, 91052 Erlangen, Germany; 5 Department of Infectious Diseases, West German Centre of Infectious Diseases, Universitätsmedizin Essen, University Duisburg-Essen, Essen, Germany; 6 Department of Pediatric Cardiac Surgery, Friedrich-Alexander University of Erlangen-Nürnberg (FAU), University Hospital Erlangen, Erlangen, Germany; 7 Department of Cardiac Surgery, Universitäts-Kinderspital Zürich, Zurich, Switzerland; 8 Department of Pediatrics II, Pediatric Nephrology, Gastroenterology, Endocrinology and Transplant Medicine, Children’s Hospital Essen, University Duisburg-Essen, Duisburg, Germany; 9 Medical Immunology Campus and German Centre for Immuntherapy (Deutsches Zentrum für Immuntherapie-DZI) Erlangen, FAU Erlangen-Nürnberg, 91054 Erlangen, Germany; 10 The Children’s Hospital of Zhejiang University School of Medicine, Hangzhou, China

**Keywords:** Allotransplantation, Plasmacytoid dendritic cells, Translational immunology, Acute inflammation, Preclinical research

## Abstract

Allograft-specific regulatory T cells (T_reg_ cells) are crucial for long-term graft acceptance after transplantation. Although adoptive T_reg_ cell transfer has been proposed, major challenges include graft-specificity and stability. Thus, there is an unmet need for the direct induction of graft-specific T_reg_ cells. We hypothesized a synergism of the immunotolerogenic effects of rapamycin (mTOR inhibition) and plerixafor (CXCR4 antagonist) for T_reg_ cell induction. Thus, we performed fully-mismatched heart transplantations and found combination treatment to result in prolonged allograft survival. Moreover, fibrosis and myocyte lesions were reduced. Although less CD3^+^ T cell infiltrated, higher T_reg_ cell numbers were observed. Noteworthy, this was accompanied by a plerixafor-dependent plasmacytoid dendritic cells-(pDCs)-mobilization. Furthermore, in vivo pDC-depletion abrogated the plerixafor-mediated T_reg_ cell number increase and reduced allograft survival. Our pharmacological approach allowed to increase T_reg_ cell numbers due to pDC-mediated immune regulation. Therefore pDCs can be an attractive immunotherapeutic target in addition to plerixafor treatment.

## Introduction

Induction and maintenance of donor-specific allo-immune tolerance is dependent on the balance of effector T cells and regulatory CD4^+^ T cells (T_reg_ cells)^1^. The latter play an essential role in the development of long-term allograft acceptance^2^ and tolerance^3^. Hence, establishing protocols for immunotherapies targeting T_reg_ cells in the setting of solid organ transplantation has gained an intense interest. Tipping this balance in favor of donor-specific regulation by an adoptive transfer of T_reg_ cells represents an attractive approach to achieve long-term allograft survival^4–6^. However, critical issues, such as isolation and storage of ex vivo expanded human T_reg_ cells, represent a significant challenge for the clinical routine application^1,7,8^. Another important problem is their potential functional instability, which might lead to an undesirable switch to a pro-inflammatory state after adoptive T_reg_ cell transfer^7^. Alternatively, the expansion of the patient's T_reg_ cells or the conversion of naïve CD4^+^ T cells into T_reg_ cells in vivo, for example by the administration of recombinant IL-2, have been proposed^9^. These in vivo expanded and/or induced T_reg_ cells are expected to be allo-educated by exposure to the allograft leading to donor allo-antigen-specific T_reg_ cells^1^. Furthermore, immunosuppressive therapies routinely applied after transplantation can hamper the development of a durable immunomodulatory effect after adoptive transfer of T_reg_ cells^10–12^.

Dendritic cells (DCs) play a pivotal role in orchestrating T cell immune responses. To ensure the balance between self-tolerance and pathogen eradication, DCs are not only able to expand effector T cells, they can also foster the differentiation of naturally occurring T_reg_ cells (nT_reg_ cells) in the thymus^13,14^ or the differentiation of naïve CD4^+^ T cells into so-called induced T_reg_ cells (iT_reg_ cells) in the periphery^15^. This property has been mainly linked to immature conventional dendritic cells (cDCs)^14,15^, which are characterized by a low-level presentation of antigenic peptides in the absence of co-stimulatory molecules. Therefore, the role of immature, and thus tolerogenic, cDCs has been tested in various murine models of autoimmune diseases^13^, such as Crohn's like ileitis^16^, autoimmune diabetes^17^ and collagen-induced arthritis (CIA)^18^, allergy^19^, but also graft versus host disease^20^ as well as transplantation^21^. These studies as well as first Phase I–II clinical trials in patients with autoimmune diseases, such as type 1 diabetes, rheumatoid arthritis or Crohn's disease^22–26^, but also in the setting of organ transplantation^27^, suggests that DCs represent a promising therapeutic alternative to conventional, unspecific immunosuppressive drugs when tolerogenic DCs are adoptively transferred. This is of special importance as the therapeutic setting with immunosuppressive drugs is often associated with severe side effects as well as limited efficacy.

Although the main function of plasmacytoid DCs (pDCs), which are a subpopulation of DCs, is the secretion of type I interferons in response to viral infections^28–32^, pDCs have been proposed to play a role in immune tolerance^28–30,33^. They are per se characterized by an altered costimulatory molecule expression profile and poor allo-stimulatory capacity when interacting with T cells. Thus, pDCs have been considered as a possible targets to induce and maintain allo-immune tolerance^33–38^. Loschko et al. demonstrated that pDCs contribute to antigen-specific peripheral immune tolerance after antigen delivery to the surface molecule sialic acid binding Ig-like lectin H (Siglec-H)^39^. This was mediated by inducing hyporesponsive CD4^+^ T cells exhibiting reduced expansion and Th1/Th17 cell plasticity^39^. Besides, pDCs have been shown to inhibit immune responses in several murine disease models including asthma, type 1 diabetes, systemic lupus erythematosus, rheumatoid arthritis, and experimental autoimmune encephalomyelitis (EAE), a murine model for multiple sclerosis, graft-versus-host disease as well as allogeneic transplant rejection by reducing pathogenic effector T cell responses or by promoting T_reg_ cells^29,40–47^.

Various immunosuppressive therapies are in clinical routine to counteract the allo-reactive inflammatory response, e.g. by fostering T_reg_ cell responses. One way is to apply calcineurin-inhibitors that may not only compromise the survival of allo-reactive effector T cells, but also negatively influence the function and survival of allo-reactive T_reg_ cells^10,11^. In contrast, the mTOR inhibitor rapamycin and its derivatives have been shown to favor the development and compartmental expansion of T_reg_ cells, while inhibiting the activation of effector T cells and B cells, both, in mice and humans^11,48–50^. Inhibitors of mTOR have not only been suggested to augment selective expansion of T_reg_ cells in vivo^51,52^, they also prevent DC-maturation and hamper the upregulation of MHC class II and co-stimulatory molecules^53^. Injection of such tolerogenic rapamycin-conditioned DCs into mice has been proposed to prolong graft survival in various transplantation models^54,55^.

Due to constant self-renewal and expansion of hematopoietic progenitor cells, the bone marrow is a rich source of premature, rather tolerogenic cells, including T cells, B cells, pDCs and to a low extent also conventional DCs and macrophages. From about 1.5% of all bone marrow-residing CD4^+^ T cells^56^, approximately one-third of them are CD4^+^FoxP3^+^ T_reg_ cells^57^. Interestingly, pDCs are enriched in the bone marrow implicating a role in maintenance of the immuntolerogenic state^28^.

Plerixafor (AMD3100) is a CXCR4 antagonist shown to disrupt the interaction of the C-X-C chemokine receptor CXCR4 with its ligand SDF1α, which is essentially produced by bone marrow stromal cells. After application of plerixafor it was noted that bone marrow-residing cells were released into the peripheral circulation^58,59^. Thus, meanwhile plerixafor is clinically used to mobilize CXCR4-expressing hematopoietic stem and progenitor cells from the bone marrow into the peripheral blood for separation and subsequent transplantation in several hematologic disorders^60^. CXCR4 is widely expressed in the hematopoietic cell compartment^61^. Hence, application of CXCR4 antagonists does not only lead to a mobilization of hematopoietic stem and progenitor cells but is accompanied by marked leukocytosis that affects all hematopoietic lineages including T_reg_ cells^60,62^. Moreover, homing into and retention of both, T_reg_ cells^56^ and DCs^63^, in the bone marrow have been proposed to be dependent on CXCR4/SDF1α interaction as well as the constitutive expression of VCAM-1 and endothelial selectins^28^. Since DCs and their progenitors express CXCR4, they will be translocated to the circulation after the application of plerixafor.

We hypothesized that the immunotolerogenic effects of the mTOR inhibitor rapamycin may synergize with the CXCR4 antagonist plerixafor to allow for a mobilization of T_reg_ cells out of the bone marrow to be enriched in a heart allograft. By applying a full-mismatch heart allograft murine model, we here provide evidence that plerixafor induced and maintained the expansion of FoxP3^+^ T_reg_ cells into the periphery and into the heart allograft. We observed that this expansion was accompanied by the recruitment of pDCs from the bone marrow into the peripheral blood, secondary lymphoid organs, and the allograft. Following this line, the in vivo depletion of pDCs led to an abrogation of the achieved prolongation of heart allograft survival by plerixafor rendering pDCs important targets for future immunotherapies in the context of transplantation as well as autoimmune diseases. Overall, we conclude that the combination of the CXCR4 antagonist plerixafor with the mTOR inhibitor rapamycin improves heart allograft survival.

## Results

### Treatment with plerixafor and rapamycin prolongs allograft survival by reducing the inflammatory allo-response

**Figure 1:**
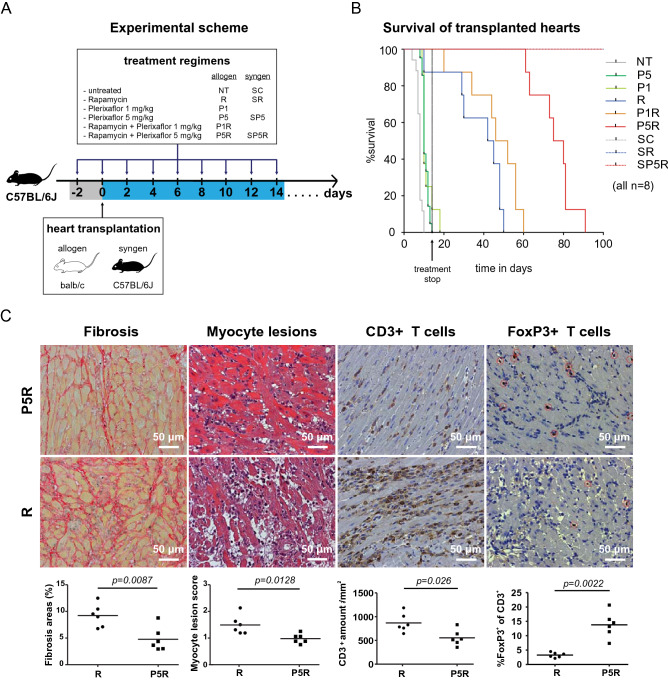
Plerixafor plus rapamycin treatment reduces the allo-inflammatory response and prolongs heart allograft survival. As outlined in panel (**A**), C57BL/6J recipients received injections with plerixafor (1 or 5 mg/kg s.c.) and/or rapamycin (0.4 mg/kg i.p.) regarding to their treatment arm two days before, immediately after HTX and following every other day until 14 days post transplantation. As indicated in the survival curves in (**B**), P5R treatment resulted in longer allograft survival compared to rapamycin-only (*p* < 0.001). Median allograft survival time in recipients from the non-treatment (NT), plerixafor (P1), plerixafor (P5), rapamycin (R) and combined treatment group P1R or P5R were 8, 10, 10, 44, 49 and 78 days, respectively. Hearts of syngeneic controls (SC, untreated; SR, rapamycin only; and SP5R, rapamycin + plerixafor) survived the whole observation period of 100 days. Survival time of heart transplants was assessed in 8 mice of each treatment group. The panels in (**C**) depict representative allograft sections recovered 14 days post-transplantation from recipients treated with plerixafor plus rapamycin (P5R, all n = 6 allografts) and rapamycin-only (R, all n = 6 allografts). Results are summarized in scatter plots (including mean) demonstrating that treatment with plerixafor plus rapamycin resulted in significantly less fibrosis (first panel, Sirius red) and less myocyte lesions (second panel, Hematoxylin Eosin). The analysis of CD3^+^ and FoxP3^+^ T cellular infiltrates (red circles) in serial sections in the third and fourth panel shows fewer CD3^+^ T cell infiltrations associated with a higher FoxP3^+^/CD3^+^ ratio for plerixafor and rapamycin treatment (cells/mm^2^, P5R). *p* values were calculated using the Gehan–Breslow–Wilcoxon test (**B**) or the Mann–Whitney-*U* test (**C**).

To investigate whether CXCR4 blockade mediates additional beneficial immune regulatory effects, we utilized an abdominal heterotopic murine heart transplantation model as previously described^64,95^ and analyzed the survival of the heart transplants by palpation according to Martins et al.^65^. The C57BL/6J recipient mice received either fully-mismatched BALB/c (allogeneic) or C57BL/6J (syngeneic) heart transplants and were treated two days before and every other day for 14 days post transplantation. As outlined in Fig. 1A, the mice either received only vehicles [no treatment, NT (allogeneic) or SC (syngeneic)], 0.4 mg/kg rapamycin + plerixafor vehicle [R (allogeneic) or SR (syngeneic)], 1 mg/kg plerixafor + rapamycin vehicle (P1), 5 mg/kg plerixafor + rapamycin vehicle (P5), 1 mg/kg plerixafor + 0.4 mg/kg rapamycin (P1R), or 5 mg/kg plerixafor + 0.4 mg/kg rapamycin [P5R (allogeneic) or SP5R (syngeneic)].

Our analysis revealed that hearts in allogeneic recipients with basic immunosuppression by rapamycin (R) demonstrated a better survival compared to vehicle-treated recipients (NT) (43.5 vs. 8 days; Fig. 1B). While the addition of low-dose plerixafor (P1R) yielded a moderate additional survival prolongation (49 vs. 43.5 days), the higher dose of plerixafor (P5R) led to a marked prolonged heart allograft survival (77.5 vs. 43.5 days, Fig. 1B). Furthermore, animals treated only with plerixafor harbored a slightly prolonged allograft survival for both, P1 (10 days) and P5 groups (10 days) compared vehicle-only controls (NT; 8 days). Thus, our data indicated a prolonged allograft survival by combining rapamycin with Plerixaflor.

To assess the transplants' general health status, we performed histological analyses. Therefore, allografts were removed 14 s post transplantation from vehicle controls (NT), plerixafor only (P5), rapamycin only (R), or rapamycin + plerixafor (P5R) treated animals and analyzed for fibrosis and myocyte lesions (Fig. 1C). We found less extensive signs of fibrosis (Fig. 1C, first panel) and a lower myocyte lesion score (Fig. 1C, second panel) in allografts of P5R animals. As expected, vehicle treated controls (NT) and plerixafor-only-treated animals (P5) showed an almost total tissue destruction (data not shown). Thus, our data revealed a better health status of the allografts upon treatment with rapamycin and plerixafor (P5R).

Furthermore, we evaluated the infiltration of CD3^+^ T cells (major drivers of allograft rejection) into the transplants by histology. Thereby, in line with less severe tissue destruction, we revealed a reduced CD3^+^ T lymphocyte infiltration after plerixafor + rapamycin (P5R) treatment compared to rapamycin-only (R) (Fig. 1C, third panel). Since mTOR inhibition might favor the expansion of CD4^+^ T_reg_ cells^11,48,66^ and these cells are a critical prerequisite for long-term allograft survival, we investigated the effect of plerixafor on the number of allograft-residing FoxP3^+^ T_reg_ cells. Therefore, we visualized them in heart allograft sections by a FoxP3 antibody as such cells are also stained by CD3. This data is represented as percentage of FoxP3^+^ cells within all CD3 cells by division of the number of FoxP3^+^ cells by the total number of CD3^+^ T cells. Surprisingly, although the CD3^+^ T cell number was reduced in the P5R group (Fig. 1C, third panel), we found higher FoxP3^+^ T_reg_ cell numbers (Fig. 1B, forth panel) compared to rapamycin-only treated animals (R). This is clearly indicated by the FoxP3^+^/CD3^+^ ratio (Fig. 1C, fourth panel). Of note, also the absolute T_reg_ cell numbers were increased by about 2.5-fold (72.8 ± 8.0 (P5R) vs. 28.1 ± 3.3 (R) T_reg_ cells/mm^2^). Collectively, our data demonstrate synergistic effects of plerixafor and rapamycin fostering heart allograft survival and reducing inflammation induced tissue destruction.

### Plerixafor treatment increased FoxP3^+^ T_reg_ cell numbers in circulation and allograft

**Figure 2:**
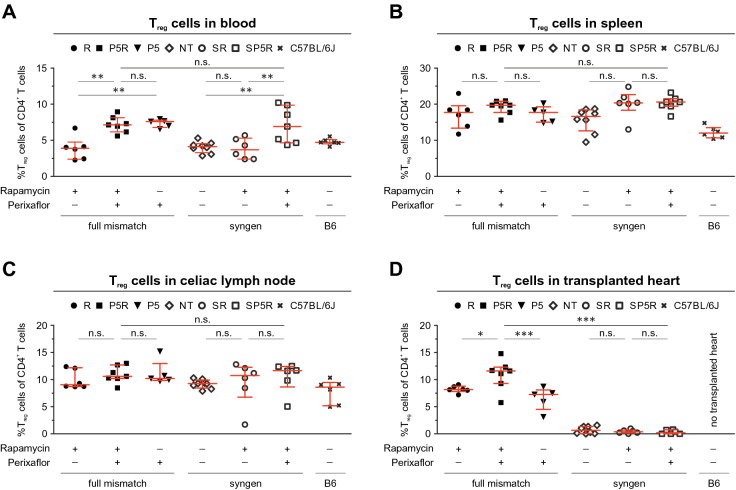
Plerixafor treatment leads to an increase of CD4^+^FoxP3^+^ T_reg_ cells in blood and the heart transplant 14 days post-transplantation. To investigate whether the increased T_reg_ cell numbers in the transplanted heart are effected by combining rapamycin and plerixafor (P5R) among the different treatment groups the content of T_reg_ cells within blood, secondary lymphoid organs and the transplanted hearts were analyzed 14 days after transplantation by flow cytometry. Single cell suspensions of blood, spleen, celiac lymph node, and the transplanted heart were generated and stained with an antibody cocktail to identify T_reg_ cells (CD3, CD4, CD19, CD25, FoxP3, Ly6G, Ter119). The scatter plot depict the percentages of T_reg_ cells (mean ± SD) within the CD4^+^ T cell population for (**A**) blood, (**B**) spleen, (**C**) celiac lymph node, and (**D**) transplanted heart (specimen from P5 n = 5, from P5R and SP5R n = 7, from NT n = 8 and all others n = 6 mice). Each data point represents the value of one mouse, all assessed 14 days after heart transplantation. Statistical analyses (One-way ANOVA with Bonferroni post-hoc test) were performed using Prism (ver. 5.01). (n.s., p > 0.05; *p ≤ 0.05; **p ≤ 0.005; ***p ≤ 0.0005).

Rapamycin can have beneficial effects on survival and proliferation of T_reg_ cells^11,50,51^ and might expand this subset^11,48,66–69^*.* To characterize this effect in our model, we analyzed T_reg_ cell numbers in blood, spleen, celiac lymph node, and the transplanted hearts 14 days post transplantation by flow cytometry. Therefore, we stained single cell suspensions using CD19, CD161b/c (NK1.1), CD3, CD4, CD25, and FoxP3 (Supplemental Fig. S1). Besides the fully mismatched heart transplantations (R, P5, and P5R), we included syngeneic control transplantations [vehicle-only (SC), rapamycin-only (SR), and rapamycin + plerixafor (SP5R)]. This comparison of allogeneic and syngeneic transplants allowed to shed light T_reg_ cells' specificity. While we found no increase of CD4^+^Foxp3^+^ Treg cell numbers in peripheral blood of transplanted versus non-transplanted C57BL/6 mice after treatment with rapamycin-only (R and SR) or vehicle-only (SC) (Fig. 2A, Supplemental Table 1), our data indicated an significant increase FoxP3^+^CD4^+^ T cells in blood by plerixafor (P5) and plerixafor + rapamycin (P5R and SP5R) (Fig. 2A, Supplemental Table 1). This increase was neither observed in spleen nor in celiac lymph nodes. Of note, syngeneic heart transplants (SC, SP5, and SP5R) did not contain T_reg_ cells (Fig. 2D, Supplemental Table 1). In contrast, infiltration of CD4^+^FoxP3^+^ T cells into the allograft was only augmented by P5R treatment in comparison plerixafor-only (P5) or rapamycin-only (R), when mice received fully mismatched heart allografts (Fig. 2D, Supplemental Table 1).

Thus, we conclude that plerixafor increased T_reg_ cell numbers in peripheral blood in our allogeneic and syngeneic heterotypic heart transplantation model. Rapamycin and plerixafor have a synergistic effect on the T_reg_ cell infiltration into fully mismatched heart transplants explaining the prolongation of heart allograft survival in the respective treatment groups.

### Plerixafor mobilize PDCA-1^+^Siglec-H^+^ pDCs into blood and celiac lymph nodes, while the concomitant rapamycin application increases infiltration into the heart allograft

**Figure 3:**
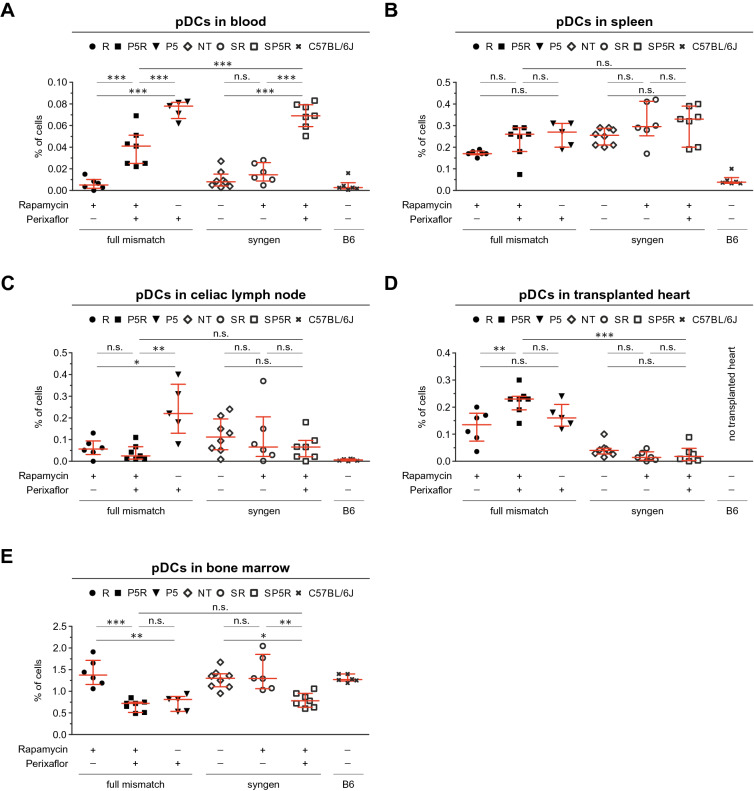
Plerixafor mobilizes pDCs from bone marrow into peripheral blood, the celiac lymph nodes and heart allograft 14 days post-transplantation. To elucidate the role of pDCs upon treatment with Plerixaflor and rapamycin in the heart transplantation setting, we investigated the presence of pDCs by flow cytometry in the peripheral blood, spleen, celiac lymph nodes, bone marrow, and samples of the left ventricle of the transplanted hearts 14 days after heterotopic allogeneic or syngeneic transplantation (this figure). For these experiments, pDCs were analyzed using a staining with CD19, CD161b/c (NK1.1), CD317 (PDCA-1, Bst-2), and Siglec-H as depicted in the Supplemental Fig. S1. Single cell suspensions of blood, spleen, celiac lymph node, and the transplanted heart were generated and stained with an antibody cocktail to identify pDCs (CD3, CD4, CD19, CD11c, CD317 (Bst-2, PDCA-1), Siglec-H, Ly6G, Ter119). The scatter plots depict the percentages (mean ± SD) of pDCs within the living cell population for (**A**) blood, (**B**) spleen, (**C**) celiac lymph node, (**D**) transplanted heart, and (**E**) bone marrow (specimen from P5 n = 5, from P5R and SP5R n = 7, from NT n = 8 and all others n = 6 mice). Each data point represents one mouse, all assessed 14 days after heart transplantation. Statistical analyses were performed using the One-way ANOVA with Bonferroni post-hoc test (n.s., p > 0.05; *p ≤ 0.05; **p ≤ 0.005; ***p ≤ 0.0005).

The application of rapamycin can lead to increased pDC numbers, which might be pro-tolerogenic by augmenting proliferation of T_regs_ cells^70–72^. Therefore, we hypothesized that the heart allograft survival prolongation by rapamycin + plerixafor is pDC mediated. Thus, we investigated pDC numbers by flow cytometry in blood, spleen, celiac lymph nodes, bone marrow, and samples of the left transplanted heart ventricle 14 days after transplantation (Fig. 3, Supplemental Table 1). pDCs were analyzed using a staining with CD19, CD161b/c (NK1.1), CD317 (PDCA-1, Bst-2), and Siglec-H, and defined as PDCA-1^+^Siglec-H^+^ (Supplemental Fig. S1).

Our data revealed higher pDC numbers in peripheral blood of plerixafor-alone (P5), but also plerixafor + rapamycin (P5R) treated animals compared to rapamycin-only (R) (Fig. 3A). A similar increase was also observed after syngeneic heart transplantation (SP5R vs. SR) demonstrating independency from the allogeneic setting. Of note, in the allogeneic setting the blood pDCs increase was less pronounced in plerixafor + rapamycin (P5R) compared plerixafor-only (P5) treated animals (Fig. 3A, Supplemental Table 1). While pDC numbers were not elevated in celiac lymph nodes (Fig. 3C, Supplemental Table 1), but in the allograft (Fig. 3D, Supplemental Table 1) within the plerixafor + rapamycin group (P5R), these cells were likely mobilized from the BM (Fig. 3E, Supplemental Table 1) by the CXCR4 blockade. In spleen no changes of pDC numbers were observed (Fig. 3B, Supplemental Table 1). Of note, plerixafor increased pDC numbers in celiac lymph nodes (Fig. 3C, Supplemental Table 1) only in the allogeneic heart transplant setting. Finally, the corresponding bone marrow specimen (Fig. 3E, Supplemental Table 1) demonstrated decreased pDC numbers after plerixafor treatment (P5, P5R, and SP5R) compared to rapamycin-only treatment (R) only in allogeneic and syngeneic heart transplant settings. We also analyzed the accumulation of pDCs in heart allografts by flow cytometry. Thereby, we found higher pDC numbers after rapamycin + plerixafor treatment (P5R) (Fig. 3D, Supplemental Table 1). In contrast, the few immune cells in syngeneic heart transplants demonstrated unchanged pDC numbers. Additionally, we also investigated other immune cell subsets, such as B cells, CD4^+^ and CD8^+^ T cells, Ly6C^high^ and Ly6C^low^ monocytes, neutrophils, as well as cDC1 and cDC2 dendritic cells, in peripheral blood, transplanted heart, splenic single cell suspensions. No changes within the frequency of these subsets, which can potentially interact with Treg cells or dampen the immune response, could be associated with the prolongation of heart allograft survival induced by rapamycin + plerixafor treatment (Supplemental Fig. S2, Supplemental Table 1).

In summary, this data demonstrate a plerixafor-mediated pDC mobilization into peripheral blood, celiac lymph nodes and heart allografts. Furthermore, we could observe a plerixafor mediated enrichment of pDCs in celiac lymph nodes in the allogenic transplantation setting.

### Depletion of PDCA-1^+^Siglec-H^+^ pDCs causes a decrease of FoxP3^+^ Treg numbers in the periphery and heart allograft abrogating the synergistic effect of plerixafor and rapamycin

**Figure 4:**
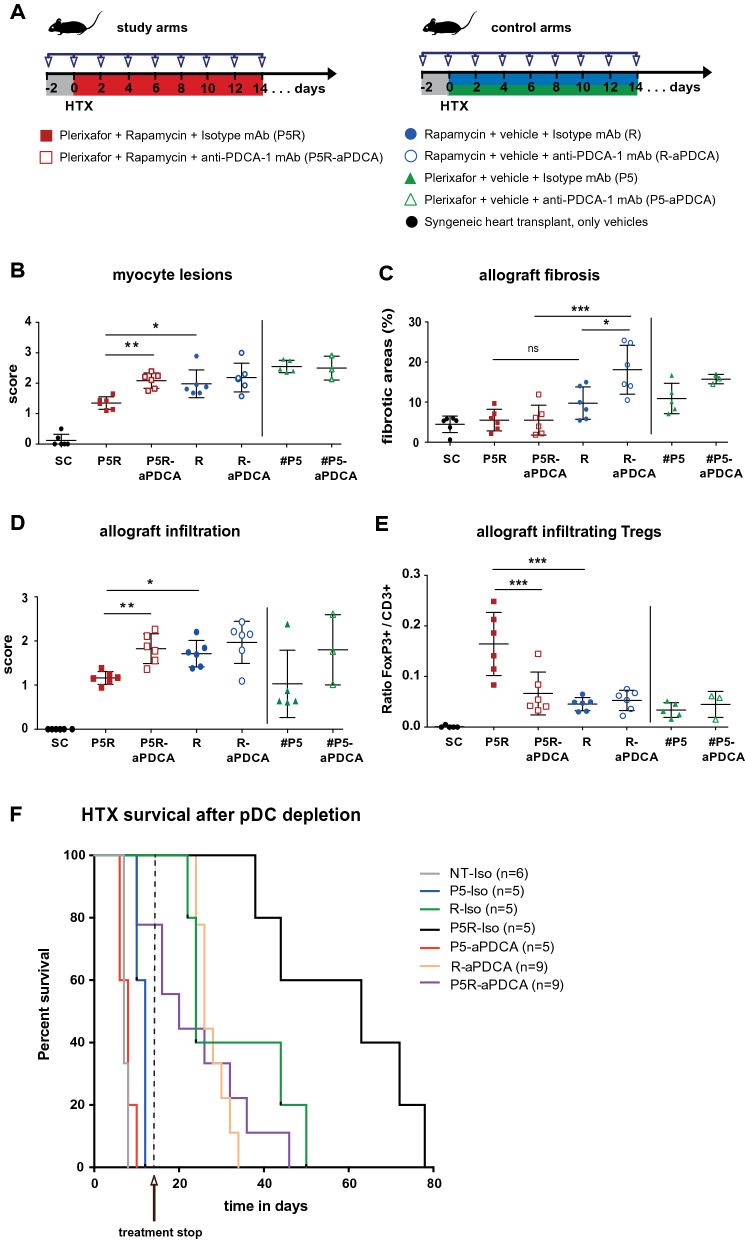
Depletion of PDCA-1^+^Siglec-H^+^pDCs results in abrogation of plerixafor mediated prolongation of heart allograft survival. To investigate the contribution of pDCs to the prolongation of allograft survival and the increase of FoxP3^+^ T_reg_ cell numbers, we performed pDCs depletion experiments in the allogeneic heart transplantation setting (outlined in **A**) and investigated the allografts for myocyte lesions (**B**), allograft fibrosis (**C**), allograft infiltrating immune cells (**D**), and T_reg_ cells (**E**) 14 days after transplantation. Example data for the stainings can be found in Supplemental Fig. S4. The scatter plots in (**B**–**E**) depict data of 3 to 6 heart transplant specimens. In these experiments, pDCs were depleted in vivo by intraperitoneally injecting a rat anti-mouse PDCA-1 mAb two days before transplantation and following every other day until 14 days post transplantation along with the general treatment. Treatment groups (study arms in red, control arms in blue and green) and time points of drug or vehicle injections for post-transplant in vivo anti-PDCA-1 depletion experiments are depicted in (**A**). Allograft sections taken from P5R treated recipients that underwent pDC-depletion (P5R-aPDCA) showed significantly higher myocyte lesion scores (**B**), a tendency of increased fibrosis (**C**) and increased cellular allograft infiltrations (**D**) compared to animals with isotype injections (P5R). The scatter plots in (**E**) demonstrate that pDC-depletion resulted in a significant decrease of allograft infiltrating CD4^+^FoxP3^+^ Treg cells determined as ratio in between Foxp3^+^ cells among CD3^+^ T cell infiltrations. (**F**) HTX survival after pDC depletion: P5R treatment with concomitant anti-PDCA-1 depletion resulted in abrogation of the prolongation of allograft survival compared to P5R treated animals that received isotype injections (P5R + anti-PDCA, n = 9 mice, median survival 20 days; P5R + Isotype, n = 5 mice, median survival 63 days; *p* < 0.01). Examined groups of allogeneic heart transplanted animals included vehicles + mAb-isotype injections (NT-Iso, n = 6), plerixafor + mAb-isotype injections (P5-Iso, n = 5), rapamycin + mAb-isotype injections (R-Iso, n = 5), plerixafor + rapamycin + mAb-isotype injections (P5R-Iso, n = 5), plerixafor + anti-PDCA mAb (P5-aPDCA, n = 5), rapamycin + anti-PDCA mAb (R-aPDCA, n = 9), plerixafor + rapamycin + anti-PDCA mAb (P5R-aPDCA, n = 9). Horizontal lines of scatter plots in (**B**–**E**) show the mean ± SD, the p value was calculated using the One-way ANOVA with Bonferroni post-hoc test (n.s., p > 0.05; *p ≤ 0.05; **p ≤ 0.005; ***p ≤ 0.0005). *SC* Syngeneic heart transplanted mice receiving only vehicles for plerixafor as well as rapamycin plus isotype mAb. ^#^Since both control groups of P5 and P5-aPDCA (green filled and open triangles) were recovered 14 days after transplantation but did undergo full rejection several days ago and were thus not beating anymore after HTX recovery these animals were excluded from statistical analysis (indicated by the vertical black line in scatter plot graphs **A**–**D**).

To investigate the contribution of pDCs to the prolongation of allograft survival and the increase of FoxP3^+^ T_reg_ cell numbers, we performed pDCs depletion experiments in the allogeneic heart transplantation setting and investigated the allografts for myocyte lesions (Fig. 4B), allograft fibrosis (Fig. 4C), allograft infiltrating immune cells (Fig. 4D), and T_reg_ cell numbers (Fig. 4E) 14 days after transplantation. We depleted pDCs by an in vivo intraperitoneal injection with anti-PDCA-1 antibodies two days before and every second day till 14 after transplantation (Fig. 4A). We treated animals as before by added an anti-PDCA-1 depletion antibody (P5R-aPDCA, R-aPDCA, P5-aPDCA) and the corresponding isotype control antibody (P5R, R, P5) (Fig. 4A). Syngeneic heart transplantations were performed in vehicles treated animals (SC). The achieved pDC depletion with a single dose of anti-PDCA-1 mAb was very effective in peripheral blood, spleen, LN and thymus, whereas bone marrow Siglec-H^+^ pDCs were not completely depleted (Supplemental Fig. S3A). Of note, tissue macrophages of spleen, lymph node, thymus, and bone marrow tissues were not affected by the treatment with the anti-PDCA-1 depletion antibody (Supplemental Fig. S3A). The pDC depletion was maintained for the whole treatment period (data not shown). Tissue macrophages (MerTK^+^) did not express PDCA-1 in our experimental setting (data not shown), but their numbers were reduced by anti-PDCA-1 treatment in the different groups (Supplemental Fig. S3B). We suspect therefore that the reduction is an indirect effect due to the depletion of the pDCs. Only a few B cells were found in the transplanted heart tissues and were mainly negative for PDCA-1 expression. Only after plerixafor only (P5-Iso) treatment, PDCA-1 expression could be detected and those cells were also reduced in numbers by anti-PDCA-1 treatment (Supplemental Fig. S3C).

Our analysis revealed that the pDC depletion in rapamycin + Perixaflor (P5R) treated mice (P5R-aPDCA1) led to a stronger cardiac allograft lymphocyte infiltration and a higher myocyte lesion score (Fig. 4B). The fibrosis of the allograft was also increased by anti-PDCA-1 treatment in the R-aPDCA1 and the P5R-aPDCA-1 groups (Fig. 4C). Furthermore, P5R + anti-PDCA-1 treated mice (P5R-aPDCA1) displayed significantly less CD4^+^FoxP3^+^ T_reg_ cells in blood and allograft (Fig. 4D,E). These results demonstrated the association of CD4^+^FoxP3^+^ T_reg_ cell enrichment with the increase of PDCA-1^+^Siglec-H^+^ pDCs.

Finally, anti-PDCA-1 depletion (P5R-aPDCA) abrogated the prolongation of allograft survival compared to P5R treated animals receiving isotype injections (P5R; 63 vs. 20 days; Fig. 4F). Rapamycin only treated animals displayed no changes in heart allograft survival with (R-aPDCA1) or without (R) pDC depletion. Of note, a slight but significant reduction in median allograft survival time was observed in P5-aPDCA1 versus isotype antibody treated P5 animals (8 vs. 12 days). Therefore, our data suggest an essential role of pDCs for the synergistic prolongation of heart allograft survival by plerixafor treatment, but are dispensable in rapamycin only treatments. Of note, we could observe a tendency of an increased IL-10 expression by pDCs within the heart tissue after treatment with rapamycin + plerixafor (P5R) compared to rapamycin treatment (R) as displayed in Supplemental Fig. S6A. Of note, nearly all IL-10 expressing cells found in the transplanted hearts could be identified as pDCs. Furthermore, we also observed a tendency in a lower type I interferon expression in the transplanted heart tissues after rapamycin + plerixafor (P5R) treatment compared to rapamycin (R) alone.

In conclusion, we found that the in vivo depletion of pDCs abrogated both, T_reg_ cell expansion as well as the plerixafor-mediated prolongation of allograft survival. Therefore, our data indicate that plerixafor is capable to reduce the severity of allograft rejection only under at least a low dosed concomitant mTOR inhibition by recruiting bone marrow-residing PDCA-1^+^Siglec-H^+^ pDCs into both, secondary lymphoid organs and the allograft.

## Discussion

The broad clinical application of adoptive T_reg_ cell immunotherapy is hindered by the risk of T_reg_ cell stability and the required consistency of purity and regulatory potency^1^. Therefore, there is still an unmet need for the direct modulation of the host's immune system. We here investigated an alternative approach to reduce allo-immune responses after allogeneic heart transplantation in mice combining the CXCR4 antagonist plerixafor with low-dose rapamycin. We observed a recruiting of pDCs from the bone marrow into the allograft and identified these cells as key players.

Here, we demonstrated a synergistic effect of plerixafor for rapamycin treatment allowing for a longer allograft survival accompanied by an amelioration of myocardial damage, fibrosis and a significant higher allograft infiltration of Foxp3^+^ T_reg_ cells (Figs. 1 and 2). plerixafor mobilized pDCs from bone marrow and their depletion reverted all positive effects of plerixafor including the prolongation of allograft survival.

pDCs and T_reg_ cells allograft infiltration upon plerixafor treatment was dependent on the fully mismatched transplant setting (Figs. 2 and 3) and therefore, we concluded its dependency on alloantigen presence. The infiltration itself might be either triggered by the alloantigen presentation in secondary lymphoid organs or by the alloimmune response in the transplant inducing expression of inflammatory chemokine ligands, such as CXCL9-11 or MIP1α^73,74^. Although CXCL12 fostered trafficking of CXCR4^+^ progenitor epithelial cells into tracheal transplants^75^, CXCL12 expression was not altered in heart allografts (data not shown). Immune cell trafficking is dependent on chemokine-chemokine receptor networks^76^. Thus, changes within the allografts may foster T_reg_ cell and pDC infiltration under mTOR inhibition as this was previously observed for T_reg_ cells in a murine islet allograft model^77^.

In experimental ischemic cardiomyopathy, immune cell migration from bone marrow (including hematopoietic progenitor cells, HSCs) improved recovery after ischemia/reperfusion injury^78,79^. In line with our data, a CXCR4 blockade fostered tissue repair after myocardial infarction due to T_reg_ cell mobilization^80^, but also speeded up the healing process^78,81^.

In line, we observed a longer allograft survival after plerixafor treatment and demonstrated further that pDC depletion resulted in reversal of these effects (Fig. 4). This suggests a certain effect of plerixafor alone synergizing with rapamycin immunosuppression. In rat liver transplantations a similar synergism of plerixafor with low-dose tacrolimus was reported and accompanied by a strong increase of allograft-residing T_reg_ cells^82^. In addition, in pancreatic islet transplantation into hyperglycemic C57BL/6 mice a CXCR4 antagonist prolonged graft survival^58^. The authors also attributed this to hematopoietic stem cells (HSCs) due to the abrogation of these effects by αCD117 antibody mediated depletion of HSCs. However, the expression of CD117 not limited to these cells as it can also be found on many other precursor cell populations including the most import pDCs precursor population, suggesting that also pDCs will be affected. The authors excluded a role of T_reg_ cells by depletion with an anti-CD25 antibody, which did not fully abrogate the prolongation of allograft survival. Rapamycin as well as plerixafor can directly influence the functionality of T_reg_ cells. Whereas rapamycin has been reported to foster T_reg_ cell function^69,83–85^, plerixafor was shown to partially reduce T_reg_ cell-mediated immunosuppression^86^.

In this study, we also investigated the role of pDCs in our murine heart transplant model as other immune cell population could not directly by associated with prolonged allograft survival by rapamycin + plerixafor (Fig. 3 and Supplemental Fig. S2. pDCs have been reported to foster the suppressive function of T_reg_ cells^34,35^. The experiments demonstrated that plerixafor not only resulted in the well-known peripheral shedding of hematopoietic stem cells^58,59^, but also of PDCA-1^+^Siglec-H^+^ pDCs. We identified an immune regulatory role of mobilized pDCs, as their anti-PDCA-1-mediated depletion resulted in a reduction of T_reg_ cells in both, blood and allograft. pDCs have been described to suppress the peripheral immune response in several murine models of auto- and allo-inflammatory disorders^13^, such as autoimmune arthritis^44^, autoimmune encephalomyelitis^40^, and graft-versus-host disease^20,43^. Whereas donor-derived cDCs have been traditionally regarded as principal instigators of allograft rejection, considerable evidence demonstrates a role for recipient’s pDCs in mediating the induction and/or maintenance of tolerance to allografts^3,34^. Abe et al. first reported a prolongation of murine cardiac allograft survival by an adoptive transfer of BM-derived pDCs^87^. In addition, Turnquist et al. observed an enrichment of antigen-specific FoxP3^+^ T_reg_ cells promoting heart allograft tolerance upon adoptive transfer of in vitro rapamycin preconditioned DCs^88^. Furthermore, pDCs participate in lupus models in peripheral tolerance by inducing T_reg_ cells^29^. Importantly, in operational tolerant pediatric liver recipients higher peripheral blood pDC numbers (compared to cDCs) have been observed^89^, while elevated myeloid cell/pDC ratios were associated with early acute cellular rejection after pediatric small bowel transplantation^90^ and late rejection after pediatric liver transplantation^91^, respectively.

There are different possibilities for the mechanism of pDC-driven induction of heart allograft-specific T_reg_ cells. They may result from a direct T_reg_ cell induction in the thymus by pDCs from the periphery, which have uptaken peripheral antigens, presenting those in the thymus promoting central tolerance^42^. Although, we observed an increased homing of pDCs into celiac lymph nodes and the cardiac allograft, we cannot specify where these interactions take place in our model. In line with these results, previous studies from Ochando et al. demonstrated homing of pDCs, which acquired donor-specific allo-antigens, to draining LNs where they induced de novo allo-antigen-specific T_reg_ cells under tolerizing conditions^47^. Furthermore, additional studies from Liu et al. identified pDCs as crucial players for establishing cardiac allograft tolerance in mice lacking the lymph node homing receptor CCR7^92^. Furthermore, pDCs might be also involved in the recruitment, differentiation, and/or functional modulation of tissue macrophages involved in tissue repair as we observed a reduction of those cells after pDC depletion while excluding a direct depletion due to lacking PDCA-1 expression (Supplemental Fig. S3B).

In summary, we demonstrate for the first time an immunomodulatory potency of plerixafor under sub-therapeutical mTOR inhibition after allogeneic heart transplantation. Combining the immunosuppressive drug with the mobilization of bone-marrow-residing pDCs allowed to reduce allograft rejection leading to prolongation of allograft survival in vivo. Here, we do not exclude a role for hematopoietic stem cells on heart allograft survival, but rather classify pDCs as an essential population for the prolonged heart allograft survival by plerixafor. We proofed the essential involvement of pDCs in the immune regulatory mechanisms in check, because their depletion resulted in an impairment of T_reg_ cell expansion and prolongation of allograft survival. Furthermore, a strong and significant exacerbation of allograft histology scores after pDC depletion occurred.

These results may impact on future clinical immunosuppressive therapies since the direct adoptive transfer of T_reg_ cells implies several disadvantages^93,94^. Therefore, it is of utmost importance to develop alternatives relying on the direct induction of donor-specific T_reg_ cells within the patient. Here, we have demonstrated that a combination of immunosuppressive drugs implementing plerixafor might be able to provide the possibility to achieve this in vivo.

## Materials and methods

### Animals

BALB/c (allogeneic) or C57BL/6J (syngeneic) mice served as heart donors and C57BL/6J as transplant recipients (all male and 8–12 weeks old). All animal experiments adhered to EU directive 2010/63/EU and were approved by the 'Regierung von Unterfranken' (approval #G1071/09) and were carried out in accordance to the ARRIVE guidelines. For anesthesia, 10% Ketamine (100 mg/kg, Dopalen) and 2% Xylazine (10 mg/kg, Rompun) was used. Metamizol (200 mg/kg) p.o. and Carprofen (5 mg/kg) s.c. served as postoperative analgesia.

### Abdominal-heterotopic cardiac transplantation model

Heterotopic intra-abdominal heart transplantation (HTX) from BALB/c or C57BL/6J to C57BL/6J recipient mice was performed as previously described^64^. As video demonstrating the whole process including a detailed method description can be found in our recent publication^95^. Allograft function was evaluated daily by palpation according to Martins et al.^65^. C57BL/6J recipients received injections with plerixafor (1 or 5 mg/kg s.c.) and/or rapamycin (0.4 mg/kg i.p.) two days before, immediately after HTX and every other day for 14 days. The subclinical dosage of rapamycin allowed to early distinguish differences in allograft survival. The treatment groups are depicted in Fig. 1A. Transplantation of C57BL/6J hearts into C57BL/6J recipients served as non-rejection controls. The role of pDCs was investigated by in vivo depletion experiments (groups see Fig. 4A). Therefore, 500 μg of either anti-PDCA-1 or an isotype control antibody was injected every second day starting two days before transplantation. Vehicle controls included 0.9% NaCl (plerixafor) and 0.2% Carboxymethylcellulose + 0.25% Tween-80 (rapamycin). All in vivo applied antibodies were endotoxin-free. The weight of all transplanted animals remained within physiological ranges as depicted in Supplemental Fig. S5.

### Reagents and antibodies

Plerixafor (AMD3100, Sigma, Germany) was prepared in 0.9% NaCl (injection volume 150 μl, s.c.). Rapamycin (Wyeth, Münster, Germany) dissolved in EtOH was diluted in 0.2% carboxymethylcellulose + 0.25% Tween80^®^ (i.p.). Anti-msPDCA-1 (for depletion, clone 927), ratIgG2b isotype control were obtained from Biolegend. Flow cytometry antibodies for CD3 (145-2C11 or eBio500A2), CD4 (RM4-5), CD8 (Clone 53-6.7), CD11b (M1/70), CD11c (HL3 or N418), CD19 (1D3), CD25 (PC61), CD45R/B220 (RA3-6B2), CD86 (GL-1), CD115 (T38-320), CD317/PDCA-1 (927), FoxP3 (MF-14 or MF-23), LAP-1 (TW7-16B4), Ly6C (HK1.4), Ly6G (1A8), Siglec-H (551), and Ly-76/Ter119 (TER-119) were purchased from BioLegend, BD Pharmingen, or eBioscience. Immunohistochemistry antibodies and isotype controls for CD3 (rabbit) and FoxP3 (FJL-16s) were obtained from Dako or eBioscience.

### Single cell isolation

Celiac LNs were recovered from paraaortic, pararenal, parapancreatic and mesenteric sites. Celiac LNs, spleen, and transplanted hearts (after flushing with cold DPBS) were teared into small pieces and digested in HBSS + Collagenase IV and DNAse I for 30–45 min as described before^96^. ACK lysis buffer was used to lyse erythrocytes. BM was flushed out with RPMI1640 + 5% FCS (Gibco, Thermo Fisher Scientific). Cell isolation was performed three hours after the last plerixafor injection.

### Flow cytometry

Single cells suspensions were blocked with anti-CD16/CD32 (2.4G2) and anti-FcγRIV (9E9) at 4 °C for 10 min. For intracellular staining of FoxP3, cells were fixed and permeabilized using the True-Nuclear Transcription Factor Buffer Set (Biolegend) according to manufacturer's instructions. Acquisition was performed on a Navio flow cytometer using Caluza 1.3 (Beckman Coulter) or a LSR Fortessa (SORP) using Diva. Data were analyzed using FlowJo 10.6.0 (TreeStar).

### Histological evaluation of the heart allografts

The allograft’s left ventricle was fixed in 10% formalin, paraffin-embedded, and sectioned (3 µm). Cellular infiltration and myocyte lesion scores were quantified as previously described^64^. In brief, myocyte lesion score 0: no lesion; 1: myocyte vacuolization; 2: focal myocyte necrosis (irregular border; fragmented sarcoplasm, debris, myocyte dropout); and 3: extensive myocyte necrosis (interstitial hemorrhage/eosinophil infiltration). The heart tissue fibrosis was investigated by Sirius red staining (acquisition: Axio 4.6, Carl Zeiss). Immunohistochemistry was performed after antigen retrieval using Tris–EDTA (pH 9) for 45 min (CD3) or citrate (pH 6) for 60 min followed by permeabilization using 0.25% Triton X-100 in PBS for 10 min (FoxP3). Primary antibody dilutions of 1 µg/ml (CD3) or 10 µg/ml (FoxP3) were applied for 30 min at RT. After washing, goat-anti-rabbit (3 μg/ml, Dianova Jackson ImmunoResearch) or goat-anti-rat (10 μg/ml BD Pharmingen) were stained for 30 min at RT as secondary antibodies. Scores and cells were assessed and calculated as average of 10 high power fields by blinded observers. T_reg_/T cell ratio was calculated by dividing FoxP3^+^ cell number/mm^2^ by CD3^+^ cell number/mm^2^ in serial sections. Two blinded observers evaluated five fields of the histological slides and an average scores were calculated.

### Statistical analysis

Data in bar graphs and scatter plots are expressed as mean ± SD and evaluated by Mann–Whitney *U* test (two groups) or One-way ANOVA with Bonferroni post-hoc test (multiple groups) using GraphPad Prism V5.01. Survival data were denoted as median survival days [P25–P75] and were analyzed using the Gehan–Breslow–Wilcoxon test. *p* values were expressed as *p < 0.05, **p < 0.01 and ***p < 0.001.

## Supplementary Information

Supplementary Information.

## Abbreviations

Ag: Antigen
APC: Antigen-presenting cell
cDCs: Conventional dendritic cells
CXCR4: C-X-C chemokine receptor type 4
FACS: Fluorescence-activated cell sorting
FoxP3: Forkhead box P3
HPF: High power field
i.p.: Intraperitoneal injection
Iso: Isotype
MST: Median survival time
mTOR: Mammalian target of rapamycin
P: Plerixafor
P1R: Plerixafor 1 mg/kg body weight plus rapamycin
P5R: Plerixafor 5 mg/kg body weight plus rapamycin
pDC: Plasmacytoid dendritic cells
PDCA-1: Phosducin A-1
R: Rapamycin
s.c.: Subcutaneous injection
Sctrl: Syngeneic control
SP5R: Syngeneic control with plerixafor 5 mg/kg body weight plus rapamycin
SR: Syngeneic vehicle control with rapamycin
SDF1α or CXCL12: Stromal cell-derived factor 1α
Siglec-H: Sialic acid binding Ig-like lectin H
Treg: Regulatory T cells

## References

[1] Safinia N, Scotta C, Vaikunthanathan T, Lechler RI, Lombardi G (2015). Regulatory T cells: Serious contenders in the promise for immunological tolerance in transplantation. Front. Immunol..

[2] Klaeske K, Lehmann S, Buttner P, Palitzsch R, Fischer J, Jawad K (2020). Identification of the immunological profile in rejection-free heart transplantation. Transpl. Immunol..

[3] Rogers NM, Isenberg JS, Thomson AW (2013). Plasmacytoid dendritic cells: No longer an enigma and now key to transplant tolerance?. Am. J. Transplant..

[4] Wood KJ, Bushell A, Hester J (2012). Regulatory immune cells in transplantation. Nat. Rev. Immunol..

[5] Riley JL, June CH, Blazar BR (2009). Human T regulatory cell therapy: Take a billion or so and call me in the morning. Immunity.

[6] Golshayan D, Jiang S, Tsang J, Garin MI, Mottet C, Lechler RI (2007). In vitro-expanded donor alloantigen-specific CD4^+^CD25^+^ regulatory T cells promote experimental transplantation tolerance. Blood.

[7] Ohkura N, Kitagawa Y, Sakaguchi S (2013). Development and maintenance of regulatory T cells. Immunity.

[8] Edozie FC, Nova-Lamperti EA, Povoleri GA, Scotta C, John S, Lombardi G (2014). Regulatory T-cell therapy in the induction of transplant tolerance: The issue of subpopulations. Transplantation.

[9] Singer BD, King LS, D'Alessio FR (2014). Regulatory T cells as immunotherapy. Front. Immunol..

[10] Presser D, Sester U, Mohrbach J, Janssen M, Kohler H, Sester M (2009). Differential kinetics of effector and regulatory T cells in patients on calcineurin inhibitor-based drug regimens. Kidney Int..

[11] Hoerning A, Kohler S, Jun C, Lu J, Fu J, Tebbe B (2012). Cyclosporin but not everolimus inhibits chemokine receptor expression on CD4^+^ T cell subsets circulating in the peripheral blood of renal transplant recipients. Clin. Exp. Immunol..

[12] Zeiser R, Nguyen VH, Beilhack A, Buess M, Schulz S, Baker J (2006). Inhibition of CD4^+^CD25^+^ regulatory T-cell function by calcineurin-dependent interleukin-2 production. Blood.

[13] Gordon JR, Ma Y, Churchman L, Gordon SA, Dawicki W (2014). Regulatory dendritic cells for immunotherapy in immunologic diseases. Front. Immunol..

[14] Gallegos AM, Bevan MJ (2004). Central tolerance to tissue-specific antigens mediated by direct and indirect antigen presentation. J. Exp. Med..

[15] Yamazaki S, Dudziak D, Heidkamp GF, Fiorese C, Bonito AJ, Inaba K (2008). CD8^+^ CD205^+^ splenic dendritic cells are specialized to induce Foxp3^+^ regulatory T cells. J. Immunol..

[16] Collins CB, Aherne CM, McNamee EN, Lebsack MD, Eltzschig H, Jedlicka P (2012). Flt3 ligand expands CD103(+) dendritic cells and FoxP3(+) T regulatory cells, and attenuates Crohn's-like murine ileitis. Gut.

[17] Kriegel MA, Rathinam C, Flavell RA (2012). Pancreatic islet expression of chemokine CCL2 suppresses autoimmune diabetes via tolerogenic CD11c^+^ CD11b^+^ dendritic cells. Proc. Natl. Acad. Sci. U.S.A..

[18] Park JE, Jang J, Choi JH, Kang MS, Woo YJ, Seong YR (2015). DC-based immunotherapy combined with low-dose methotrexate effective in the treatment of advanced CIA in mice. J. Immunol. Res..

[19] Escobar A, Aguirre A, Guzman MA, Gonzalez R, Catalan D, Acuna-Castillo C (2014). Tolerogenic dendritic cells derived from donors with natural rubber latex allergy modulate allergen-specific T-cell responses and IgE production. PLoS One.

[20] Scroggins SM, Olivier AK, Meyerholz DK, Schlueter AJ (2013). Characterization of regulatory dendritic cells that mitigate acute graft-versus-host disease in older mice following allogeneic bone marrow transplantation. PLoS One.

[21] Li H, Shi B (2015). Tolerogenic dendritic cells and their applications in transplantation. Cell. Mol. Immunol..

[22] Phillips BE, Garciafigueroa Y, Engman C, Trucco M, Giannoukakis N (2019). Tolerogenic dendritic cells and T-regulatory cells at the clinical trials crossroad for the treatment of autoimmune disease; emphasis on type 1 diabetes therapy. Front. Immunol..

[23] Phillips BE, Garciafigueroa Y, Trucco M, Giannoukakis N (2017). Clinical tolerogenic dendritic cells: Exploring therapeutic impact on human autoimmune disease. Front. Immunol..

[24] Benham H, Nel HJ, Law SC, Mehdi AM, Street S, Ramnoruth N (2015). Citrullinated peptide dendritic cell immunotherapy in HLA risk genotype-positive rheumatoid arthritis patients. Sci. Transl. Med..

[25] Jauregui-Amezaga A, Cabezon R, Ramirez-Morros A, Espana C, Rimola J, Bru C (2015). Intraperitoneal administration of autologous tolerogenic dendritic cells for refractory Crohn's disease: A phase I study. J. Crohns Colitis.

[26] Ten Brinke A, Hilkens CM, Cools N, Geissler EK, Hutchinson JA, Lombardi G (2015). Clinical use of tolerogenic dendritic cells-harmonization approach in European collaborative effort. Mediat. Inflamm..

[27] Ochando J, Ordikhani F, Jordan S, Boros P, Thomson AW (2020). Tolerogenic dendritic cells in organ transplantation. Transpl. Int..

[28] Swiecki M, Colonna M (2015). The multifaceted biology of plasmacytoid dendritic cells. Nat. Rev. Immunol..

[29] Swiecki M, Colonna M (2010). Unraveling the functions of plasmacytoid dendritic cells during viral infections, autoimmunity, and tolerance. Immunol. Rev..

[30] Reizis B (2019). Plasmacytoid dendritic cells: Development, regulation, and function. Immunity.

[31] Musumeci A, Lutz K, Winheim E, Krug AB (2019). What makes a pDC: Recent advances in understanding plasmacytoid DC development and heterogeneity. Front. Immunol..

[32] Webster B, Assil S, Dreux M (2016). Cell–cell sensing of viral infection by plasmacytoid dendritic cells. J. Virol..

[33] Waisman A, Lukas D, Clausen BE, Yogev N (2017). Dendritic cells as gatekeepers of tolerance. Semin. Immunopathol..

[34] Podesta MA, Cucchiari D, Ponticelli C (2015). The diverging roles of dendritic cells in kidney allotransplantation. Transplant. Rev. (Orlando)..

[35] van Kooten C, Lombardi G, Gelderman KA, Sagoo P, Buckland M, Lechler R (2011). Dendritic cells as a tool to induce transplantation tolerance: Obstacles and opportunities. Transplantation.

[36] Martin P, Del Hoyo GM, Anjuere F, Arias CF, Vargas HH, Fernandez LA (2002). Characterization of a new subpopulation of mouse CD8alpha^+^ B220^+^ dendritic cells endowed with type 1 interferon production capacity and tolerogenic potential. Blood.

[37] Colonna M, Trinchieri G, Liu YJ (2004). Plasmacytoid dendritic cells in immunity. Nat. Immunol..

[38] Boor PP, Metselaar HJ, Jonge S, Mancham S, van der Laan LJ, Kwekkeboom J (2011). Human plasmacytoid dendritic cells induce CD8(+) LAG-3(+) Foxp3(+) CTLA-4(+) regulatory T cells that suppress allo-reactive memory T cells. Eur. J. Immunol..

[39] Loschko J, Heink S, Hackl D, Dudziak D, Reindl W, Korn T (2011). Antigen targeting to plasmacytoid dendritic cells via Siglec-H inhibits Th cell-dependent autoimmunity. J. Immunol..

[40] Bailey-Bucktrout SL, Caulkins SC, Goings G, Fischer JA, Dzionek A, Miller SD (2008). Cutting edge: Central nervous system plasmacytoid dendritic cells regulate the severity of relapsing experimental autoimmune encephalomyelitis. J. Immunol..

[41] de Heer HJ, Hammad H, Soullie T, Hijdra D, Vos N, Willart MA (2004). Essential role of lung plasmacytoid dendritic cells in preventing asthmatic reactions to harmless inhaled antigen. J. Exp. Med..

[42] Hadeiba H, Lahl K, Edalati A, Oderup C, Habtezion A, Pachynski R (2012). Plasmacytoid dendritic cells transport peripheral antigens to the thymus to promote central tolerance. Immunity.

[43] Hadeiba H, Sato T, Habtezion A, Oderup C, Pan J, Butcher EC (2008). CCR9 expression defines tolerogenic plasmacytoid dendritic cells able to suppress acute graft-versus-host disease. Nat. Immunol..

[44] Jongbloed SL, Benson RA, Nickdel MB, Garside P, McInnes IB, Brewer JM (2009). Plasmacytoid dendritic cells regulate breach of self-tolerance in autoimmune arthritis. J. Immunol..

[45] Kang HK, Liu M, Datta SK (2007). Low-dose peptide tolerance therapy of lupus generates plasmacytoid dendritic cells that cause expansion of autoantigen-specific regulatory T cells and contraction of inflammatory Th17 cells. J. Immunol..

[46] Nikolic T, Welzen-Coppens JM, Leenen PJ, Drexhage HA, Versnel MA (2009). Plasmacytoid dendritic cells in autoimmune diabetes—Potential tools for immunotherapy. Immunobiology.

[47] Ochando JC, Homma C, Yang Y, Hidalgo A, Garin A, Tacke F (2006). Alloantigen-presenting plasmacytoid dendritic cells mediate tolerance to vascularized grafts. Nat. Immunol..

[48] Zeiser R, Leveson-Gower DB, Zambricki EA, Kambham N, Beilhack A, Loh J (2008). Differential impact of mammalian target of rapamycin inhibition on CD4^+^CD25^+^Foxp3^+^ regulatory T cells compared with conventional CD4^+^ T cells. Blood.

[49] Delgoffe GM, Pollizzi KN, Waickman AT, Heikamp E, Meyers DJ, Horton MR (2011). The kinase mTOR regulates the differentiation of helper T cells through the selective activation of signaling by mTORC1 and mTORC2. Nat. Immunol..

[50] Hoerning A, Wilde B, Wang J, Tebbe B, Jing L, Wang X (2015). Pharmacodynamic monitoring of mammalian target of rapamycin inhibition by phosphoflow cytometric determination of p70S6 kinase activity. Transplantation.

[51] McMahon G, Weir MR, Li XC, Mandelbrot DA (2011). The evolving role of mTOR inhibition in transplantation tolerance. J. Am. Soc. Nephrol..

[52] Biswas M, Sarkar D, Kumar SR, Nayak S, Rogers GL, Markusic DM (2015). Synergy between rapamycin and FLT3 ligand enhances plasmacytoid dendritic cell-dependent induction of CD4^+^CD25^+^FoxP3^+^ Treg. Blood.

[53] Sukhbaatar N, Hengstschlager M, Weichhart T (2016). mTOR-mediated regulation of dendritic cell differentiation and function. Trends Immunol..

[54] Taner T, Hackstein H, Wang Z, Morelli AE, Thomson AW (2005). Rapamycin-treated, alloantigen-pulsed host dendritic cells induce Ag-specific T cell regulation and prolong graft survival. Am. J. Transplant..

[55] Li X, Li JJ, Yang JY, Wang DS, Zhao W, Song WJ (2012). Tolerance induction by exosomes from immature dendritic cells and rapamycin in a mouse cardiac allograft model. PLoS One.

[56] Zhao E, Xu H, Wang L, Kryczek I, Wu K, Hu Y (2012). Bone marrow and the control of immunity. Cell. Mol. Immunol..

[57] Zou L, Barnett B, Safah H, Larussa VF, Evdemon-Hogan M, Mottram P (2004). Bone marrow is a reservoir for CD4^+^CD25^+^ regulatory T cells that traffic through CXCL12/CXCR4 signals. Cancer Res..

[58] Fiorina P, Jurewicz M, Vergani A, Petrelli A, Carvello M, D'Addio F (2011). Targeting the CXCR4-CXCL12 axis mobilizes autologous hematopoietic stem cells and prolongs islet allograft survival via programmed death ligand 1. J. Immunol..

[59] Broxmeyer HE, Orschell CM, Clapp DW, Hangoc G, Cooper S, Plett PA (2005). Rapid mobilization of murine and human hematopoietic stem and progenitor cells with AMD3100, a CXCR4 antagonist. J. Exp. Med..

[60] Bilgin YM, de Greef GE (2016). Plerixafor for stem cell mobilization: The current status. Curr. Opin. Hematol..

[61] Loetscher M, Geiser T, O'Reilly T, Zwahlen R, Baggiolini M, Moser B (1994). Cloning of a human seven-transmembrane domain receptor, LESTR, that is highly expressed in leukocytes. J. Biol. Chem..

[62] Couban S, Wong PC, Schultz KR (2019). The case for plerixafor to replace filgrastim as the optimal agent to mobilize peripheral blood donors for allogeneic hematopoietic cell transplantation. Exp. Hematol..

[63] Nakano H, Lyons-Cohen MR, Whitehead GS, Nakano K, Cook DN (2017). Distinct functions of CXCR4, CCR2, and CX3CR1 direct dendritic cell precursors from the bone marrow to the lung. J. Leukoc. Biol..

[64] Wu K, Turk TR, Rauen U, Su S, Feldkamp T, de Groot H (2011). Prolonged cold storage using a new histidine-tryptophan-ketoglutarate-based preservation solution in isogeneic cardiac mouse grafts. Eur. Heart J..

[65] Martins PN (2008). Assessment of graft function in rodent models of heart transplantation. Microsurgery.

[66] Hoerning A, Koss K, Datta D, Boneschansker L, Jones CN, Wong IY (2011). Subsets of human CD4(+) regulatory T cells express the peripheral homing receptor CXCR3. Eur. J. Immunol..

[67] Fraser H, Safinia N, Grageda N, Thirkell S, Lowe K, Fry LJ (2018). A rapamycin-based GMP-compatible process for the isolation and expansion of regulatory T cells for clinical trials. Mol. Ther. Methods Clin. Dev..

[68] Strauss L, Whiteside TL, Knights A, Bergmann C, Knuth A, Zippelius A (2007). Selective survival of naturally occurring human CD4^+^CD25^+^Foxp3^+^ regulatory T cells cultured with rapamycin. J. Immunol..

[69] Battaglia M, Stabilini A, Roncarolo MG (2005). Rapamycin selectively expands CD4^+^CD25^+^FoxP3^+^ regulatory T cells. Blood.

[70] Rosborough BR, Hackstein H, Turnquist HR (2014). A window into immunosuppressant immunoregulation: Recipient conversion to rapamycin increases potentially tolerogenic immune cells. Kidney Int..

[71] Cao W, Manicassamy S, Tang H, Kasturi SP, Pirani A, Murthy N (2008). Toll-like receptor-mediated induction of type I interferon in plasmacytoid dendritic cells requires the rapamycin-sensitive PI(3)K-mTOR-p70S6K pathway. Nat. Immunol..

[72] Boor PP, Metselaar HJ, Mancham S, van der Laan LJ, Kwekkeboom J (2013). Rapamycin has suppressive and stimulatory effects on human plasmacytoid dendritic cell functions. Clin. Exp. Immunol..

[73] Melter M, Exeni A, Reinders ME, Fang JC, McMahon G, Ganz P (2001). Expression of the chemokine receptor CXCR3 and its ligand IP-10 during human cardiac allograft rejection. Circulation.

[74] Melter M, McMahon G, Fang J, Ganz P, Briscoe DM (1999). Current understanding of chemokine involvement in allograft transplantation. Pediatr. Transplant..

[75] Gomperts BN, Belperio JA, Rao PN, Randell SH, Fishbein MC, Burdick MD (2006). Circulating progenitor epithelial cells traffic via CXCR4/CXCL12 in response to airway injury. J. Immunol..

[76] Nourshargh S, Alon R (2014). Leukocyte migration into inflamed tissues. Immunity.

[77] Zhang N, Schroppel B, Lal G, Jakubzick C, Mao X, Chen D (2009). Regulatory T cells sequentially migrate from inflamed tissues to draining lymph nodes to suppress the alloimmune response. Immunity.

[78] Ziff OJ, Bromage DI, Yellon DM, Davidson SM (2018). Therapeutic strategies utilizing SDF-1alpha in ischaemic cardiomyopathy. Cardiovasc. Res..

[79] Doring Y, Pawig L, Weber C, Noels H (2014). The CXCL12/CXCR4 chemokine ligand/receptor axis in cardiovascular disease. Front. Physiol..

[80] Wang Y, Dembowsky K, Chevalier E, Stuve P, Korf-Klingebiel M, Lochner M (2019). C-X-C motif chemokine receptor 4 blockade promotes tissue repair after myocardial infarction by enhancing regulatory T cell mobilization and immune-regulatory function. Circulation.

[81] Proulx C, El-Helou V, Gosselin H, Clement R, Gillis MA, Villeneuve L (2007). Antagonism of stromal cell-derived factor-1alpha reduces infarct size and improves ventricular function after myocardial infarction. Pflugers Arch..

[82] Okabayashi T, Cameron AM, Hisada M, Montgomery RA, Williams GM, Sun Z (2011). Mobilization of host stem cells enables long-term liver transplant acceptance in a strongly rejecting rat strain combination. Am. J. Transplant..

[83] Battaglia M, Stabilini A, Migliavacca B, Horejs-Hoeck J, Kaupper T, Roncarolo MG (2006). Rapamycin promotes expansion of functional CD4^+^CD25^+^FOXP3^+^ regulatory T cells of both healthy subjects and type 1 diabetic patients. J. Immunol..

[84] Golovina TN, Mikheeva T, Brusko TM, Blazar BR, Bluestone JA, Riley JL (2011). Retinoic acid and rapamycin differentially affect and synergistically promote the ex vivo expansion of natural human T regulatory cells. PLoS One.

[85] Singh K, Kozyr N, Stempora L, Kirk AD, Larsen CP, Blazar BR (2012). Regulatory T cells exhibit decreased proliferation but enhanced suppression after pulsing with sirolimus. Am. J. Transplant..

[86] Wang L, Wang Z, Han R, Samanta A, Ge G, Levin LS (2020). Donor bone-marrow CXCR4^+^ Foxp3^+^ T-regulatory cells are essential for costimulation blockade-induced long-term survival of murine limb transplants. Sci. Rep..

[87] Abe M, Wang Z, de Creus A, Thomson AW (2005). Plasmacytoid dendritic cell precursors induce allogeneic T-cell hyporesponsiveness and prolong heart graft survival. Am. J. Transplant..

[88] Turnquist HR, Raimondi G, Zahorchak AF, Fischer RT, Wang Z, Thomson AW (2007). Rapamycin-conditioned dendritic cells are poor stimulators of allogeneic CD4^+^ T cells, but enrich for antigen-specific Foxp3^+^ T regulatory cells and promote organ transplant tolerance. J. Immunol..

[89] Mazariegos GV, Zahorchak AF, Reyes J, Ostrowski L, Flynn B, Zeevi A (2003). Dendritic cell subset ratio in peripheral blood correlates with successful withdrawal of immunosuppression in liver transplant patients. Am. J. Transplant..

[90] Gupta A, Ashokkumar C, Ningappa M, Sun Q, Higgs BW, Snyder S (2010). Elevated myeloid: Plasmacytoid dendritic cell ratio associates with early acute cellular rejection in pediatric small bowel transplantation. Transplantation.

[91] Gupta A, Kumar CA, Ningappa M, Sun Q, Higgs BW, Snyder S (2009). Elevated myeloid: Plasmacytoid dendritic cell ratio associates with late, but not early, liver rejection in children induced with rabbit anti-human thymocyte globulin. Transplantation.

[92] Liu X, Mishra P, Yu S, Beckmann J, Wendland M, Kocks J (2011). Tolerance induction towards cardiac allografts under costimulation blockade is impaired in CCR7-deficient animals but can be restored by adoptive transfer of syngeneic plasmacytoid dendritic cells. Eur. J. Immunol..

[93] Hansmann L, Schmidl C, Kett J, Steger L, Andreesen R, Hoffmann P (2012). Dominant Th2 differentiation of human regulatory T cells upon loss of FOXP3 expression. J. Immunol..

[94] Waldmann H, Hilbrands R, Howie D, Cobbold S (2014). Harnessing FOXP3^+^ regulatory T cells for transplantation tolerance. J. Clin. Investig..

[95] Yin D, Fu J, Allabauer I, Witzke O, Rong S, Hoerning A (2021). Blood circuit reconstruction in an abdominal mouse heart transplantation model. J. Vis. Exp..

[96] Lehmann CHK, Baranska A, Heidkamp GF, Heger L, Neubert K, Luhr JJ (2017). DC subset-specific induction of T cell responses upon antigen uptake via Fcgamma receptors in vivo. J. Exp. Med..

[97] Wang, X., Fu, J., Wilde, B., Lu, J., Zhu, J., Kribben, A., Witzke, O. & Hoerning, A. CXCR4-SDF1α blockade reduces the severity of murine heart allograft rejection [abstract #994]. *Am. J. Transplant.* **15**(suppl 3) (2015).

[98] Fu J, Wahlbuhl M, Wang X, Wilde B, Jing L, Kribben A, Hoyer P, Lehmann CHK, Dudziak D, Witzke O, Hoerning A (2017). CXCR4-SDF1 alpha blockade reduces the severity of murine heart allograft rejection. Eur. J. Immunol..

