# Supplementary material for: CXCR4 blockade reduces the severity of murine heart allograft rejection by plasmacytoid dendritic cell-mediated immune regulation
Source: Sci Rep. 2021 Dec 10;11:23815. doi: 10.1038/s41598-021-03115-z (PMC8664946; doi:10.1038/s41598-021-03115-z)
Supplement: Supplementary file 1 — Supplementary Information. [file 41598_2021_3115_MOESM1_ESM.pdf]

Supplemental figure S1: Treg and pDC gating strategy

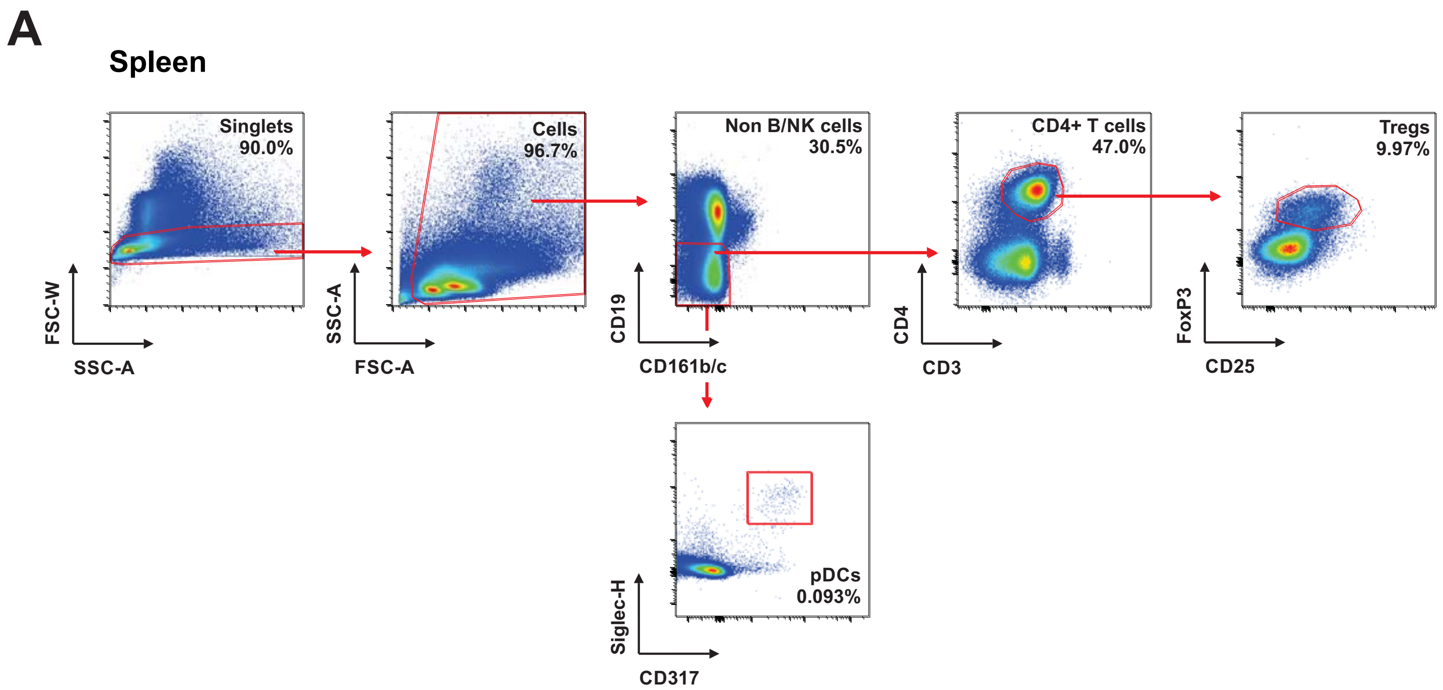

Supplemental Figure S1 *Gating strategy to identify CD4+ T regulatory cells and pDCs*

Gating strategy of splenocytes depicting flow cytometry dot blots.

Supplemental Figure S2: Rapamycin and/or plerixafor treatment influence immune cell subset distribution.

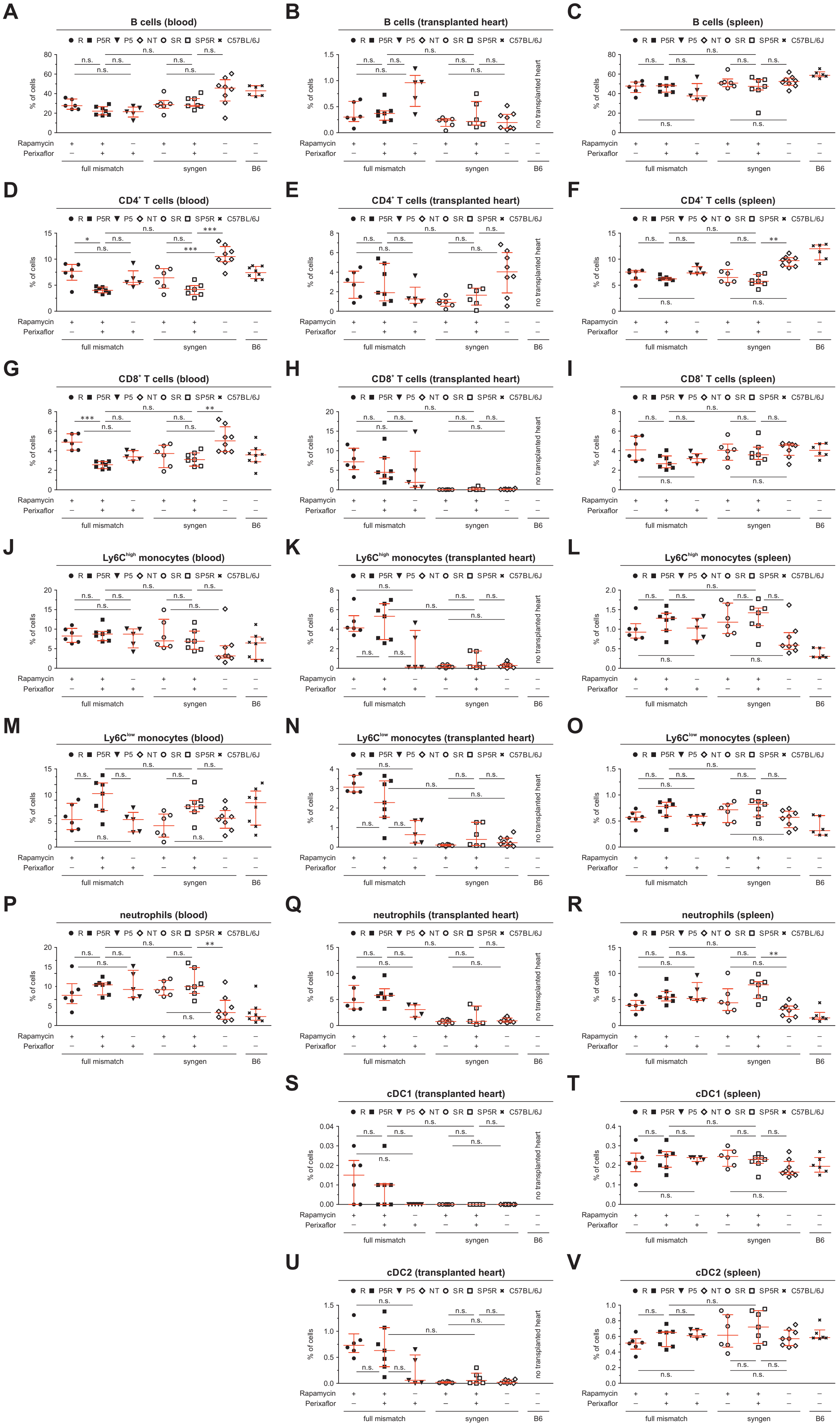

Supplemental Figure S2 *Treatment with rapamycin and/or plerixafor influenced different immune cell subsets in blood, spleen, and the transplanted heart.*

To elucidate immune cell subset changes by the treatment with Plerixaflor and Rapamycin in the heart transplantation setting, we investigated the changes of subset frequencies of B cells (A-C), $CD4^+$ (D-F) and $CD8^+$ T cells (G-I), $Ly6C^{high}$ (J-L) and $Ly6C^{low}$ monocytes (M-O), neutrophils (P-R), cDC1 (S-T) as well as cDC2 dendritic cells (U-V) by flow cytometry in the peripheral blood (A, D, G, J, M, and P), the transplanted hearts (B, E, H, K, N, Q, S, and U), and spleen samples (C, F, I, L, O, R, T, and V) 14 days after heterotopic allogeneic or syngeneic transplantation. Single cell suspensions of blood, spleen, and the transplanted heart were generated and stained with an antibody cocktail to identify the named subsets (CD3, CD4, CD19, CD11b, CD11c, CD25, CD45R (B220), CD115, CD317 (Bst-2, PDCA-1), Siglec-H, Ly6C, Ly6G, Ter119). The scatter plots depict the percentages (mean±SD) of the named subset within the living cell population for blood, transplanted heart, and spleen (specimen from P5 n=5, from P5R and SP5R n=7, from NT n=8 and all others n=6 mice). Each data point represents one mouse, all assessed 14 days after heart transplantation. Statistical analyses were performed using the One-way ANOVA with Bonferroni post-hoc test (n.s., $p>0.05$; *, $p\leq0.05$; **, $p\leq0.005$; *** $p\leq0.0005$).

Supplemental Figure S3: anti-PDCA-1 treatment results in the depletion of pDCs, but does only indirectly reduce tissue macrophage numbers and midly influence the B cells.

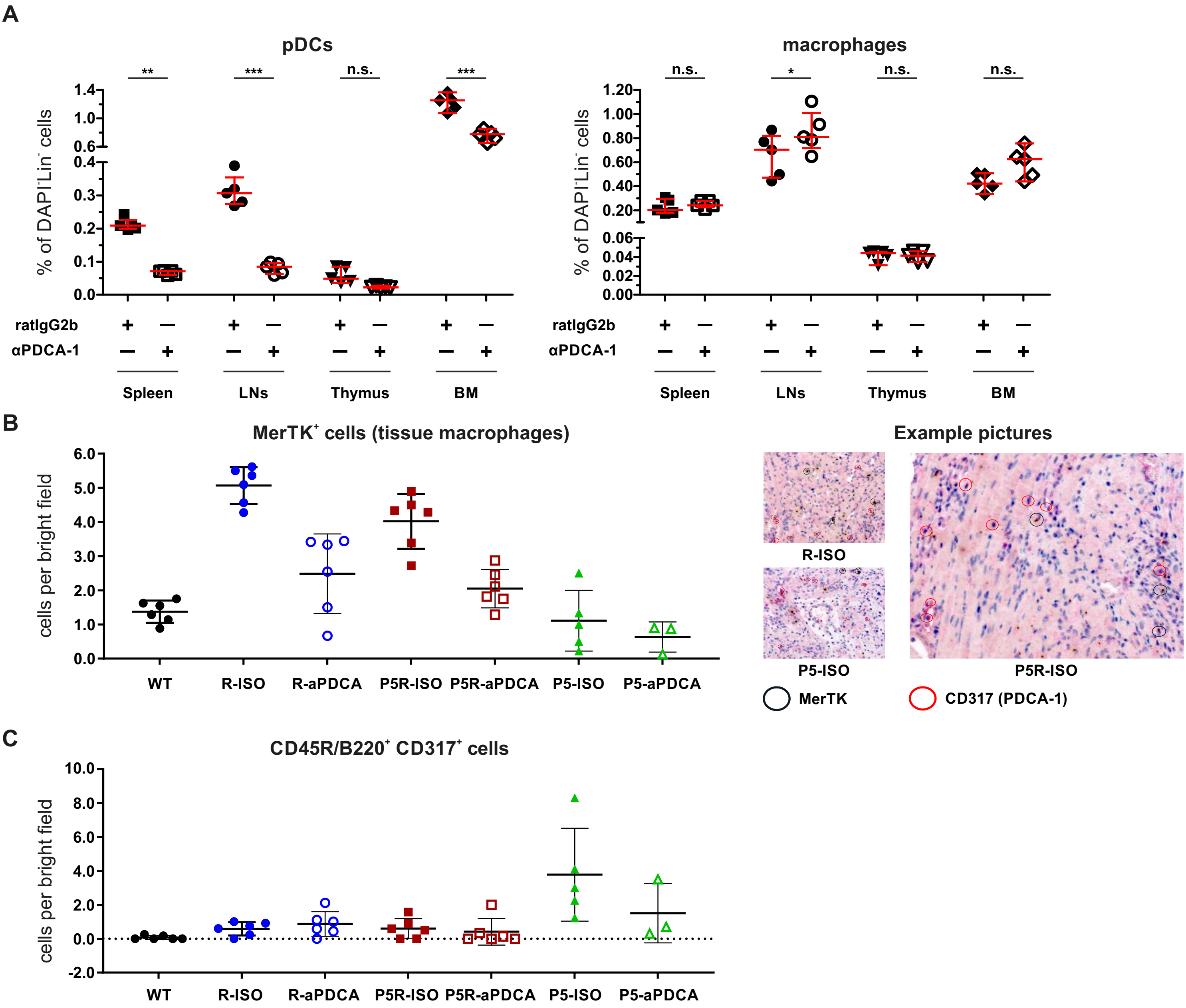

Supplemental Figure S3 *Treatment with anti-PDCA1 mAb results in pDC depletion in periphery, but not in bone marrow. This treatment also reduced tissue macrophage numbers. B cells express PDCA-1 after single plerixafor treatment and their numbers are reduced by anti-PDCA-1 treatment.*

A. In order to assess pDC depletion efficiency, we analyzed pDC numbers by flow cytometry of spleen, lymph nodes, thymus and the bone marrow 2 hours after intraperitoneal treatment with 500 µg of anti-PDCA1 or the respective isotype control (each n=5). (Lin- = negative for CD3, CD49b(DX5), CD90.2, CD161b/c(NK1.1), Ly6G, Ter119). Statistical analyses were performed using the One-way ANOVA with Bonferroni post-hoc test (n.s., $p>0.05$; *, $p\leq0.05$; **, $p\leq0.005$; *** $p\leq0.0005$). **B**. Scatter blots MerTK+ tissue macrophage numbers in heart tissue slides for the different treatment groups with or without anti-PDCA-1 treatment. WT are sliced from not transplanted heart tissues. Including example pictures (right panel) **C**. Scatter blots of CD45R/B220^{+}PDCA-1^{+} B cells in heart tissue slides for the different treatment groups with or without anti-PDCA-1 treatment. WT are sliced from not transplanted heart tissues.

Supplemental Figure S4: Example stainings for aPDCA-1 treatment

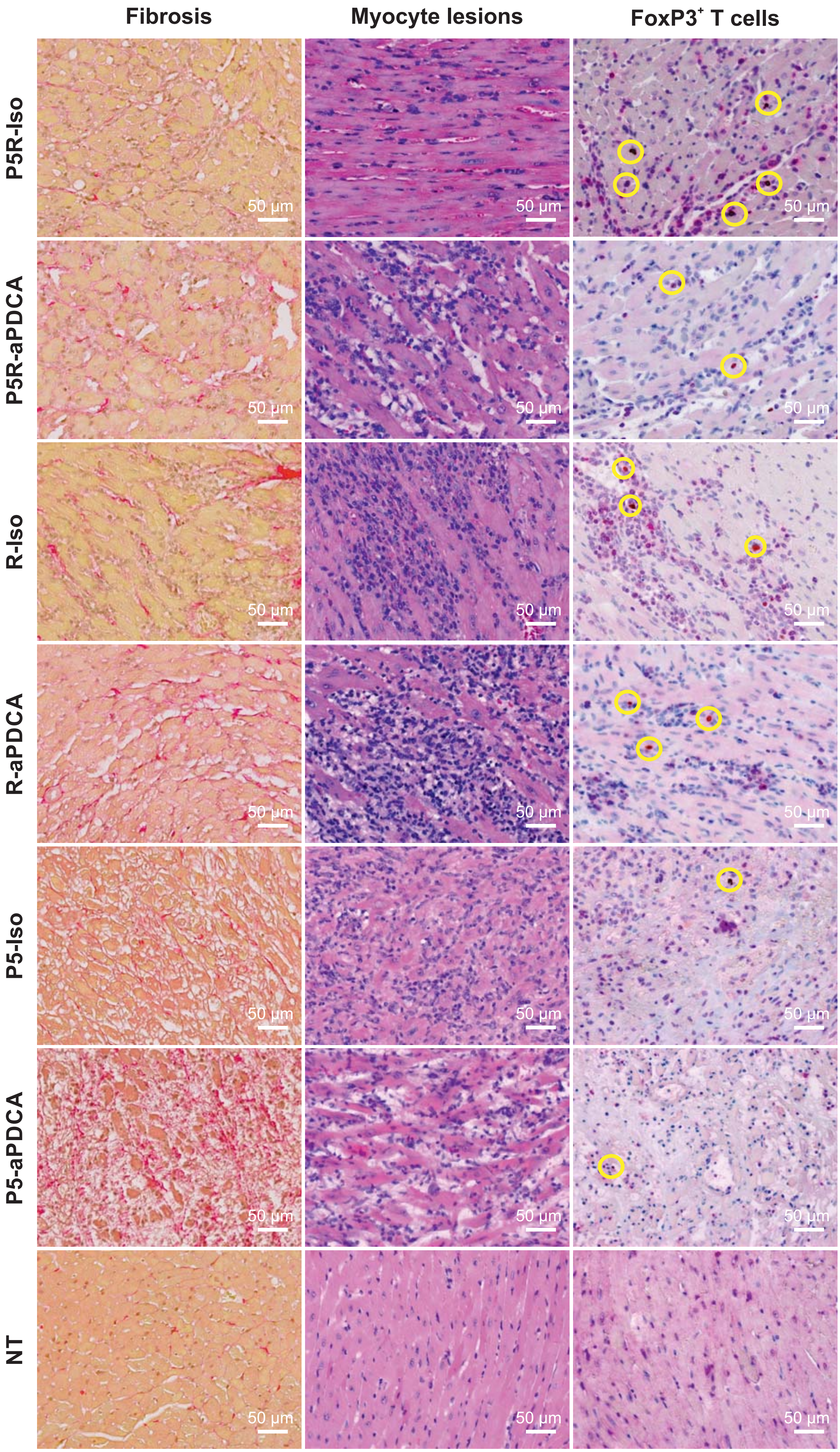

Supplemental Figure S4 *Treatment with anti-PDCA1 mAb results in deterioration of cardiac allograft histology*

Depicted are representative data related to Figure 4 showing Sirius red (to evaluate development of fibrosis, left column), HE (to evaluate myocyte lesions, middle column) and immunohistochemistry stainings for CD3 and FoxP3 (right column) in cardiac transplants harvested 14 days after transplantation (P5R-Iso, P5R-aPDCA, R-Iso, R-aPDCA, syngeneic non-treated animals) or after complete rejection exhibiting no detectable palpable beating of the heart (usually around 10 days after Tx, see also Figure 4).

Supplemental Figure S5: Weight data for transplanted mice within the different treatment groups

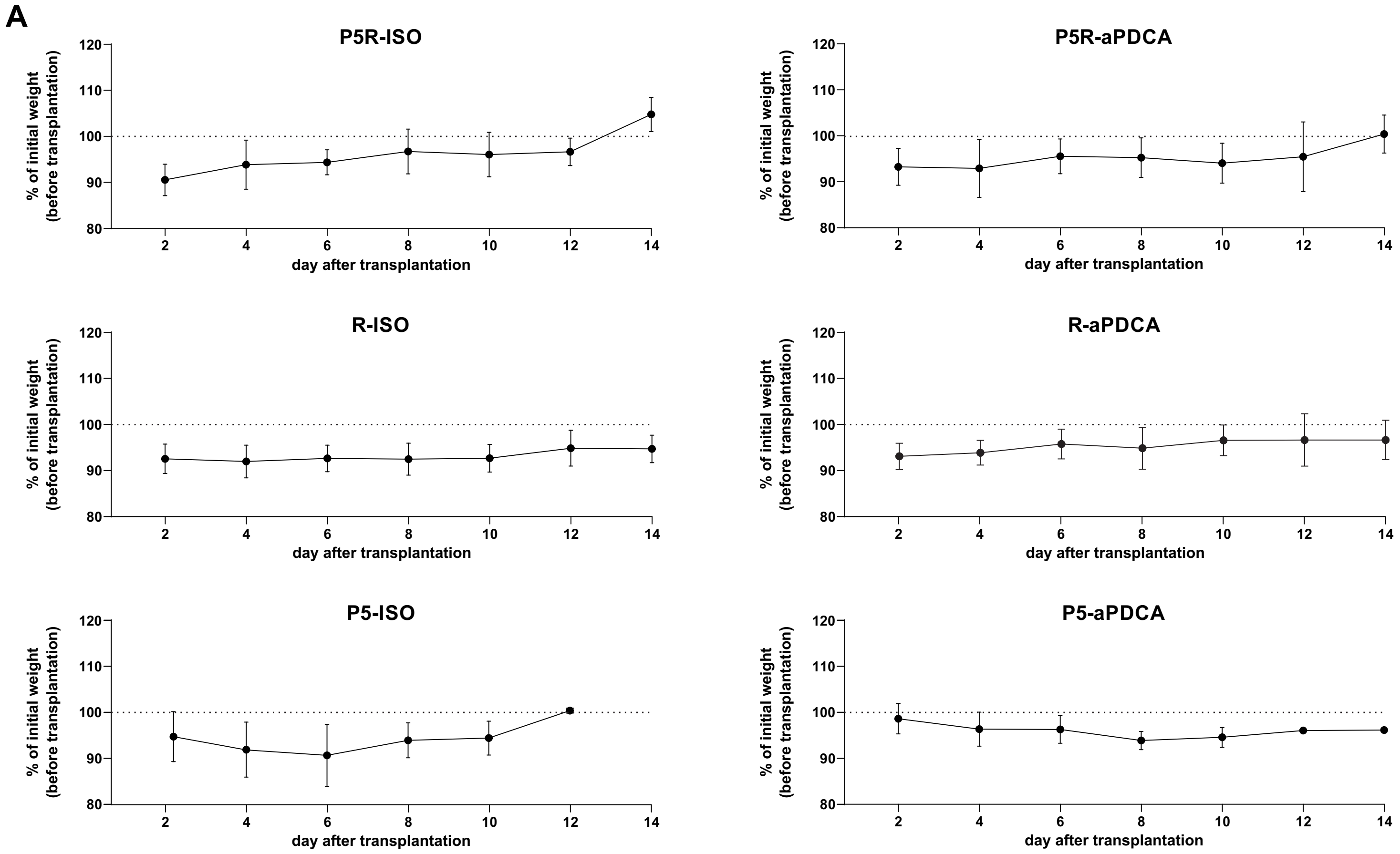

Supplemental Figure S5 *Mouse weight data for the different treatment groups with or without anti-PDCA-1 treatment display only changes within the expected physiological range*

A. Animal weight was documented and is presented as percentage of the initial weight before transplantation. Mice weight was documented for 14 days during the treatments. Curves are discontinued, if mice were euthanized due to transplant failure.

Supplemental Figure S6: IL10 is mainly expressed by pDCs and IFNα is reduced by P5R treatment

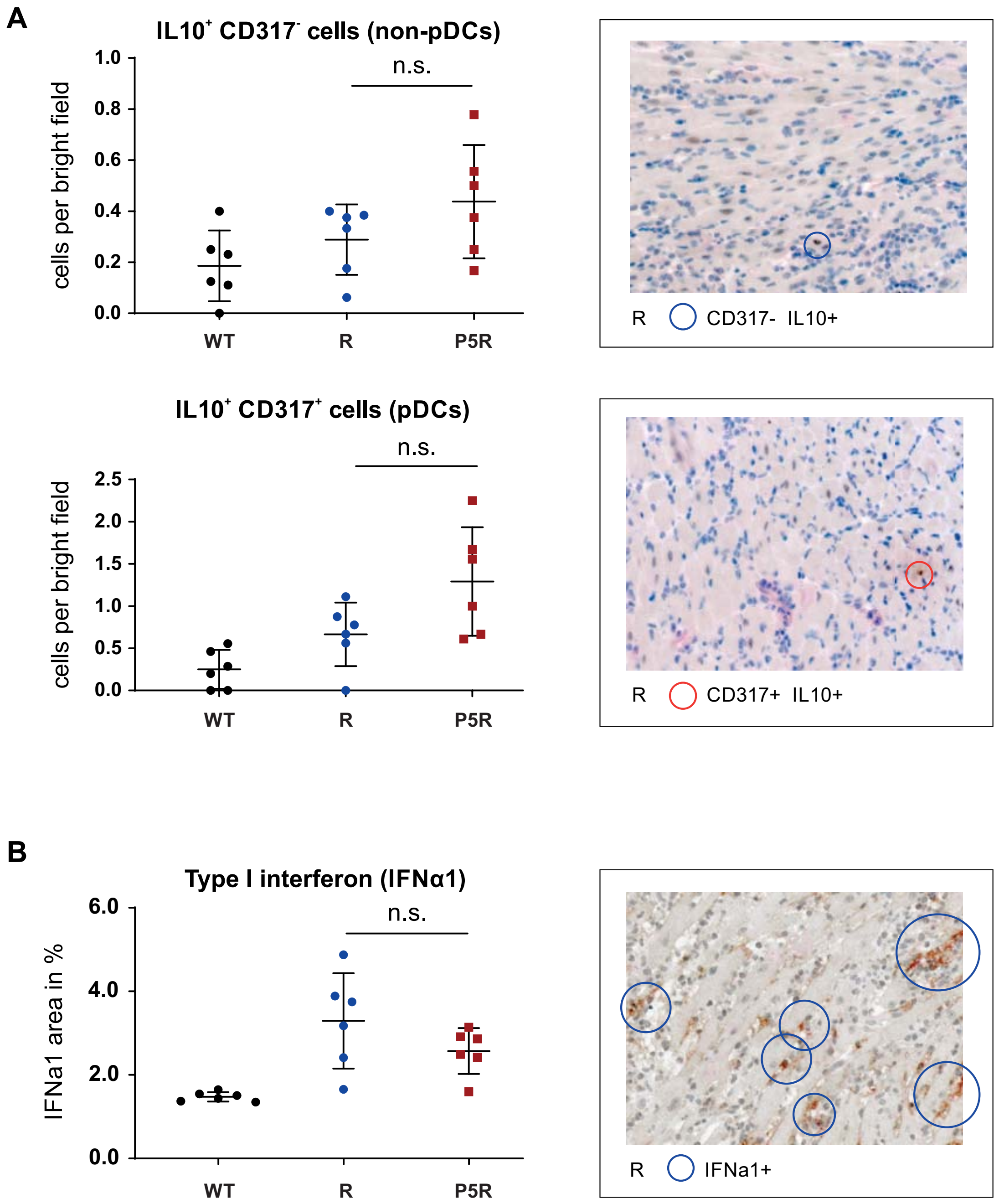

Supplemental Figure S6 *IL10 is mainly expressed by pDCs and rapamycin+plerixafor treatment reduced type I interferon (IFNα1) levels*

A. Scatter blots and representative microscopy pictures of co-stainings of PDCA-1 (CD317) and IL-10 for not transplanted heart slices (WT), rapamycin treated (R) or rapamycin+plerixafor treated animals. Presented are IL10^{+} non pDCs (meaning CD317^{-}) and IL10+ pDCs (meaning CD317^{+}). **B**. Densitometry analysis of IFNα1 positive area of the tissue slices presented as scatter blots for the not transplanted heart slices (WT), rapamycin treated (R) or rapamycin+plerixafor treated animals including a representative microscopy picture for the IFNα1 staining.

Treg Cells (%Treg of CD4+ T cells)

	R<br>(n=6)	P5R<br>(n=7)	P5<br>(n=5)	NT<br>(n=8)	SR<br>(n=6)	SP5R<br>(n=7)	C57Bl/6<br>(n=6)
**Blood**	3.890% (2.388%-4.763%)	7.130% (6.160%-8.120%)	7.580% (6.775%-7.790%)	4.115% (3.278%-4.530%)	3.690% (2.389%-5.290%)	6.900% (4.650%-9.860%)	4.710% (4.500%-5.118%)
**Spleen**	17.70% (13.35%-19.55%)	19.70% (17.70%-20.80%)	17.70% (15.00%-19.25%)	16.60% (12.60%-18.38%)	20.35% (18.33%-22.63%)	20.60% (19.40%-21.50%)	11.90% (10.60%-13.43%)
**celiac lymph node**	9.045% (8.718%-12.18%)	10.60% (10.20%-12.70%)	10.20% (9.840%-12.95%)	9.260 (8.435%-9.915%)	10.75% (6.758%-12.28%)	11.65% (8.640%-12.43%)	8.545% (5.113%-9.415%)
**transplanted heart**	8.170% (7.823%-8.790%)	11.60% (9.290%-12.30%)	7.250% (4.510%-8.100%)	0.655% (0.075%-1.348%)	0.365% (0.165%-0.693%)	0.185% (0.0%-0.733%)	-

pDCs (% of cells)

	R<br>(n=6)	P5R<br>(n=7)	P5<br>(n=5)	NT<br>(n=8)	SR<br>(n=6)	SP5R<br>(n=7)	C57Bl/6<br>(n=6)
**Blood**	0.0050% (0.0020%-0.0101%)	0.0410% (0.0250%-0.0510%)	0.0780% (0.0665%-0.0815%)	0.0080% (0.0041%-0.0151%)	0.0087% (0.0145%-0.0258%)	0.0690% (0.0590%-0.0793%)	0.0026% (0.0014%-0.0072%)
**Spleen**	0.170% (0.165%-0.183%)	0.260% (0.180%-0.290%)	0.270% (0.200%-0.310%)	0.255% (0.210%-0.288%)	0.295% (0.253%-0.413%)	0.330% (0.200%-0.390%)	0.038% (0.033%-0.060%)
**celiac lymph node**	0.057% (0.032%-0.094%)	0.025% (0.010%-0.067%)	0.220% (0.130%-0.355%)	0.112% (0.054%-0.195%)	0.066% (0.022%-0.205%)	0.066% (0.021%-0.096%)	0.0062% (0.0020%-0.0103%)
**transplanted heart**	0.135% (0.074%-0.178%)	0.230% (0.190%-0.240%)	0.160% (0.130%-0.210%)	0.040% (0.027%-0.048%)	0.015% (0.004%-0.035%)	0.018% (0.002%-0.048%)	-
**bone marrow**	1.375% (1.158%-1.715%)	0.720% (0.510%-0.750%)	0.535% (0.810%-0.880%)	1.300% (1.105%-1.405%)	1.295% (1.060%-1.853%)	0.780% (0.630%-0.950%)	1.270% (1.235%-1.400%)

B cells (% of cells)

	R<br>(n=6)	P5R<br>(n=7)	P5<br>(n=5)	NT<br>(n=8)	SR<br>(n=6)	SP5R<br>(n=7)	C57Bl/6<br>(n=6)
**Blood**	27.67% (24.04%-34.57%)	22.11% (18.35%-26.63%)	21.55% (16.11%-26.32%)	45.70% (32.51%-54.25%)	28.78% (24.97%-32.96%)	28.01% (27.01%-35.00%)	52.89% (37.79%-48.10%)
**Spleen**	47.75% (41.15%-51.90%)	48.03% (41.58%-49.04%)	37.74% (33.86%-50.28%)	52.45% (48.63%-55.98%)	50.82% (47.15%-55.04%)	47.45% (43.74%-54.33%)	58.73% (57.27%-62.32%)
**transplanted heart**	0.300% (0.215%-0.603%)	0.370% (0.240%-0.420%)	0.960% (0.505%-1.100%)	0.195% (0.085%-0.353%)	0.240% (0.123%-0.268%)	0.215% (0.148%-0.600%)	-

CD4+ T cells (% of cells)

	R<br>(n=6)	P5R<br>(n=7)	P5<br>(n=5)	NT<br>(n=8)	SR<br>(n=6)	SP5R<br>(n=7)	C57Bl/6<br>(n=6)
**Blood**	7.680% (5.950%-8.983%)	4.020% (3.530%-4.630%)	5.090% (5.550%-7.755%)	10.49% (9.495%-12.42%)	6.425% (4.415%-8.193%)	4.180% (3.220%-4.920%)	7.410% (6.268%-8.600%)
**Spleen**	7.530% (5.998%-7.845%)	6.220% (5.970%-6.510%)	7.360% (7.185%-8.550%)	9.715% (8.645%-10.29%)	6.845% (5.573%-8.010%)	5.760% (5.270%-7.060%)	12.00% (9.843%-12.73%)
**transplanted heart**	2.965% (1.325%-4.105%)	1.900% (1.060%-4.900%)	1.260% (0.081%-2.645%)	4.035% (1.873%-6.000%)	0.895% (0.515%-1.213%)	1.645% (0.628%-2.363%)	-

CD8+ T cells (% of cells)

	R<br>(n=6)	P5R<br>(n=7)	P5<br>(n=5)	NT<br>(n=8)	SR<br>(n=6)	SP5R<br>(n=7)	C57Bl/6<br>(n=6)
**Blood**	4.880% (4.063%-5.738%)	2.570% (2.160%-2.900%)	3.380% (3.025%-4.015%)	5.010% (3.920%-6.453%)	3.715% (2.273%-4.560%)	3.090% (2.430%-3.780%)	3.575% (2.875%-4.095%)
**Spleen**	4.080% (3.025%-5.483%)	2.670% (2.250%-3.460%)	3.190% (2.780%-3.695%)	4.550% (3.523%-4.683%)	4.035% (3.018%-4.675%)	3.580% (3.110%-4.360%)	4.035% (3.455%-4.723%)
**transplanted heart**	7.165% (5.178%-10.65%)	4.490% (2.970%-8.210%)	1.910% (0.635%-9.870%)	0.105% (0.073%-0.190%)	0.050% (0.038%-0.065%)	0.075% (0.038%-0.435%)	-

Ly6C(high) monocytes (% of cells)

	R<br>(n=6)	P5R<br>(n=7)	P5<br>(n=5)	NT<br>(n=8)	SR<br>(n=6)	SP5R<br>(n=7)	C57Bl/6<br>(n=6)
**Blood**	3.300% (2.648%-4.080%)	3.500% (2.820%-3.770%)	3.490% (2.085%-4.025%)	1.260% (1.045%-2.303%)	2.800% (2.210%-5.013%)	2.760% (1.930%-3.800%)	2.585% (0.908%-3.203%)
**Spleen**	0.925% (0.773%-1.143%)	1.280% (0.970%-1.410%)	1.030% (0.730%-1.280%)	0.590% (0.485%-0.913%)	1.180% (0.890%-1.668%)	1.420% (1.090%-1.540%)	0.305% (0.278%-0.523%)
**transplanted heart**	3.070% (2.825%-3.678%)	2.280% (1.530%-3.390%)	0.640% (0.205%-1.365%)	0.225% (0.070%-0.460%)	0.105% (0.063%-0.135%)	0.395% (0.088%-1.273%)	-

Ly6C(low) monocytes (% of cells)

	R<br>(n=6)	P5R<br>(n=7)	P5<br>(n=5)	NT<br>(n=8)	SR<br>(n=6)	SP5R<br>(n=7)	C57Bl/6<br>(n=6)
**Blood**	0.880% (0.560%-1.400%)	1.710% (1.170%-2.050%)	0.880% (0.485%-1.110%)	0.925% (0.603%-1.170%)	0.685% (0.318%-1.058%)	1.290% (1.130%-1.490%)	1.420% (0.703%-1.793%)
**Spleen**	0.570% (0.485%-0.673%)	0.780% (0.590%-0.860%)	0.590% (0.435%-0.600%)	0.570% (0.373%-0.688%)	0.715% (0.470%-0.828%)	0.820% (0.580%-0.890%)	0.315% (0.230%-0.605%)
**transplanted heart**	4.130% (3.785%-5.385%)	5.320% (2.940%-6.610%)	0.120% (0.110%-3.875%)	0.280% (0.093%-0.445%)	0.135% (0.093%-0.380%)	0.295% (0.093%-1.765%)	-

Neutrophils (% of cells)

	R (n=6)	P5R (n=7)	P5 (n=5)	NT (n=8)	SR (n=6)	SP5R (n=7)	C57Bl/6 (n=6)
**Blood**	23.20% (16.79%-32.16%)	31.25% (23.54%-32.72%)	27.68% (21.45%-42.42%)	9.970% (4.840%-19.34%)	27.52% (23.23%-34.65%)	29.90% (24.66%-44.50%)	6.915% (4.033%-12.52%)
**Spleen**	3.885% (2.913%-4.833%)	5.440% (4.740%-6.570%)	4.850% (5.200%-8.270%)	3.120% (1.733%-3.760%)	4.380% (2.898%-7.078%)	7.830% (5.220%-8.430%)	1.480% (1.195%-2.538%)
**transplanted heart**	4.420% (3.178%-7.710%)	5.785% (4.810%-7.058%)	3.065% (1.613%-3.970%)	0.910% (0.670%-1.438%)	0.775% (0.420%-1.075%)	0.820% (0.365%-3.740%)	-

cDC1 (% of cells)

	R (n=6)	P5R (n=7)	P5 (n=5)	NT (n=8)	SR (n=6)	SP5R (n=7)	C57Bl/6 (n=6)
**Spleen**	0.220% (0.168%-0.263%)	0.250% (0.190%-0.270%)	0.240% (0.220%-0.240%)	0.165% (0.153%-0.220%)	0.245% (0.195%-0.278%)	0.230% (0.210%-0.240%)	0.195% (0.165%-0.240%)
**transplanted heart**	0.015% (0.0%-0.023%)	0.010% (0.0%-0.010%)	0.0% (0.0%-0.0%)	0.0% (0.0%-0.0%)	0.0% (0.0%-0.0%)	0.0% (0.0%-0.0%)	-

cDC2 (% of cells)

	R (n=6)	P5R (n=7)	P5 (n=5)	NT (n=8)	SR (n=6)	SP5R (n=7)	C57Bl/6 (n=6)
**Spleen**	0.515% (0.438%-0.570%)	0.650% (0.470%-0.660%)	0.610% (0.590%-0.685%)	0.570% (0.483%-0.680%)	0.615% (0.463%-0.878%)	0.720% (0.510%-0.930%)	0.585% (0.578%-0.683%)
**transplanted heart**	0.730% (0.593%-0.950%)	0.630% (0.320%-1.070%)	0.060% (0.010%-0.545%)	0.020% (0.0%-0.065%)	0.020% (0.008%-0.033%)	0.055% (0.008%-0.195%)	-

Supplemental Table 1: Cell frequencies after heart transplantion in different organs (namely blood, spleen, celiac lymph node, transplanted heart, and bone marrow). Displayed are the median frequencies of Treg cells (% of CD4+ T cells) or cell frequencies of total cells (for pDCs, B cells, $CD4^+$ T cells, $CD8^+$ T cells, $Ly6C^{high}$ monocytes, $Ly6C^{low}$ monocytes, neutrophils, cDC1 and cDC2. The table contains the median values and the corresponding interquartile range in brackets. The corresponding scatter plots can be found in the figures 2 and 3 as well as supplementary figure S2.
